# Self-organized and directed branching results in optimal coverage in developing dermal lymphatic networks

**DOI:** 10.1038/s41467-023-41456-7

**Published:** 2023-09-21

**Authors:** Mehmet Can Uçar, Edouard Hannezo, Emmi Tiilikainen, Inam Liaqat, Emma Jakobsson, Harri Nurmi, Kari Vaahtomeri

**Affiliations:** 1 Institute of Science and Technology Austria (IST Austria), Am Campus 1, 3400 Klosterneuburg, Austria; 2 Translational Cancer Medicine Research Program, University of Helsinki, Biomedicum Helsinki, Haartmaninkatu 8, 00290 Helsinki, Finland; 3 Wihuri Research Institute, Biomedicum Helsinki, Haartmaninkatu 8, 00290 Helsinki, Finland

**Keywords:** Body patterning, Lymphangiogenesis, Computational biophysics

## Abstract

Branching morphogenesis is a ubiquitous process that gives rise to high exchange surfaces in the vasculature and epithelial organs. Lymphatic capillaries form branched networks, which play a key role in the circulation of tissue fluid and immune cells. Although mouse models and correlative patient data indicate that the lymphatic capillary density directly correlates with functional output, i.e., tissue fluid drainage and trafficking efficiency of dendritic cells, the mechanisms ensuring efficient tissue coverage remain poorly understood. Here, we use the mouse ear pinna lymphatic vessel network as a model system and combine lineage-tracing, genetic perturbations, whole-organ reconstructions and theoretical modeling to show that the dermal lymphatic capillaries tile space in an optimal, space-filling manner. This coverage is achieved by two complementary mechanisms: initial tissue invasion provides a non-optimal global scaffold via self-organized branching morphogenesis, while VEGF-C dependent side-branching from existing capillaries rapidly optimizes local coverage by directionally targeting low-density regions. With these two ingredients, we show that a minimal biophysical model can reproduce quantitatively whole-network reconstructions, across development and perturbations. Our results show that lymphatic capillary networks can exploit local self-organizing mechanisms to achieve tissue-scale optimization.

## Abstract (web summary)

The lymphatic system is a transport network that controls immune response and tissue fluid circulation in the body. Here the authors combine experiment and theory to reveal that developing lymphatic capillary networks exploit complementary branching strategies to optimize tissue coverage.

## Introduction

Tubular epithelial and endothelial organs play key roles in the transport and exchange of fluids. For efficient function, these organs need to have a high surface-to-volume ratio (e.g., lungs and kidney) and/or even coverage of large tissue volumes (blood and lymphatic vasculature), which can be achieved by complex branched structures. The organ function and tissue environment set challenges to the regulatory mechanisms of branching morphogenesis. Although the molecular players controlling branching morphogenesis vary substantially from one organ to the next, several common cellular events and gene regulatory network motifs have been identified for branching^1,2^. From a biophysical perspective, different models of branching morphogenesis have been proposed in the past^3–5^, ranging from stereotypic fractal-like dynamics such as in the mammalian lung^6^, which allows for optimal gas transport and exchange, to a self-organized process relying on local cues such as local tubule density^7–9^. Although such self-organization from local cues provides a simple mechanism to tile space, it has been shown to do so at the expense of efficiency, showing large spatial fluctuations and poor space-filling properties^9^. Beyond such mechanisms for generating tree-like structures, several works, in particular in plant and animal vasculature which often form complex loopy networks, have concentrated on rationalizing their final geometry and topology, with an emphasis on potential optimality in terms of homeostatic function^10–14^. Furthermore, numerous theoretical models have been proposed to describe the formation of vascular networks, based on both coarse-grained differential equations^15–18^, and using discrete simulation frameworks at the cellular scale^19–21^ as well as “hybrid” approaches combining (sub)cellular- and tissue-scale dynamics^22^. However, how the dynamical mechanisms of complex network growth relate to their final homeostatic structures and functions remain poorly understood.

The lymphatic vasculature forms a branched network, which is characterized by blind-ended lymphatic capillaries (the treetop) that act as a site for tissue fluid and leukocyte entry. From the lymphatic capillaries, tissue fluid-derived lymph and leukocytes run into lymphatic collector vessels (the stalk) and thereafter to a series of lymph nodes, before entering the blood circulation at the subclavian vein. Most of the lymphatic vessels (LVs) grow via branching morphogenesis (i.e., lymphangiogenesis), which proceeds from the stalk to the treetop direction^23,24^. Multiple mouse models and correlative patient data demonstrate that the lymphatic capillary density and network maturation are essential for efficient lymphatic function^23^. However, how optimal is the coverage provided by lymphatic capillaries, and what are the mechanisms ensuring such coverage, remain poorly characterized at the molecular and cellular level.

To study the mechanisms of branching morphogenesis of lymphatic capillaries, we chose the widely used model system of mouse ear pinna dermal lymphatic capillaries. The flat, quasi-two-dimensional, dermal tissue of the ear pinna allows whole network imaging and, thus, the reconstruction of the full developmental process of lymphatic capillary network formation. Here we show, by a combination of morphometric timepoint analyses, lineage tracing, genetic perturbations, quantitative reconstructions and theoretical modeling that developing lymphatic capillary networks exploit local self-organizing mechanisms to achieve tissue-scale optimization.

## Results

### Quantitative modeling of morphogenesis of the mouse ear pinna dermal lymphatic capillary network

**Fig. 1:**
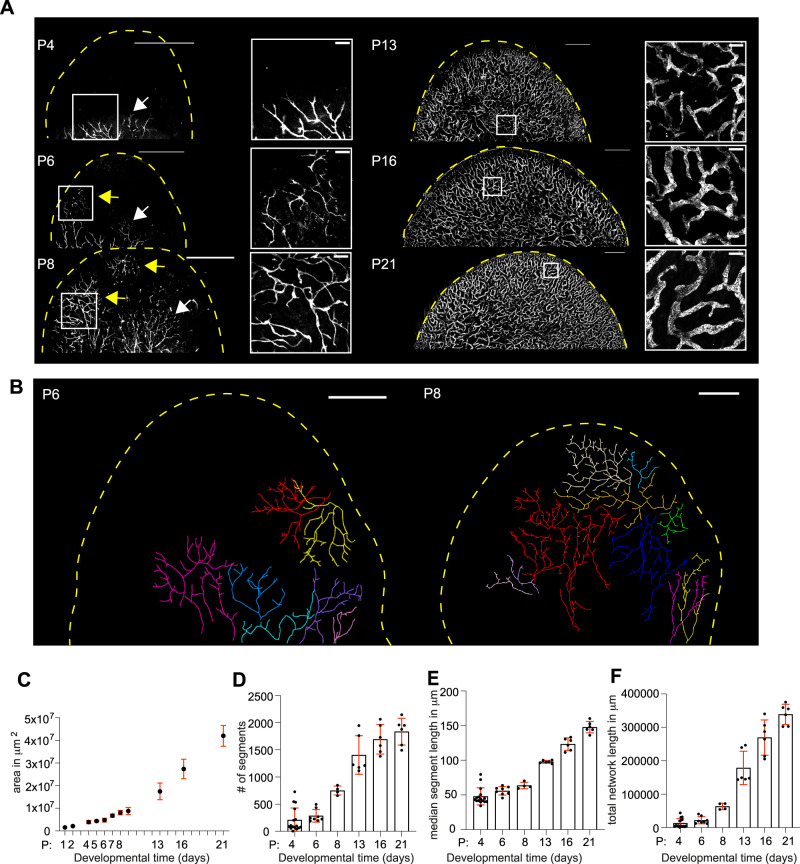
Characterization of postnatal morphogenesis of dermal lymphatic vessel network. **A** Mouse ear pinna ventral dermis stained for LYVE1 at postnatal day (P) 4, 6, 8, 13, 16, and 21. The dashed yellow line indicates the ear pinna boundary. The boxed regions are shown as magnified images. The white arrows indicate the LV trees that invade the dermis from the stalk of the ear pinna, whereas the yellow arrows indicate LV trees that invade the ventral dermis from the tip of the ear pinna. Scale bars are 1 mm in the overview and 100 μm in magnified images. See also Supplementary Movies 1–9. **B** Manual tracing of the lymphatic endothelial sub-trees growing on the superficial ventral dermis in P6 and P8 ear pinna. Each sub-tree is indicated with a unique color. Images represent *n* = 5 P6 and *n* = 5 P8 ear pinna, representing 5 mice each. See Supplementary Fig. 1F for additional samples and Supplementary Movies 2, 3, and 5. Scale bars are 500 μm. **C** Graph shows mean +/− SD of the mouse ear pinna area. Number of analyzed ear pinna for P1 *n* = 2 (in 1 mouse), P2 *n* = 2 (1 mouse), P4 *n* = 17 (9 mice), P5 *n* = 4 (2 mice), P6 *n* = 10 (5 mice), P7 *n* = 4 (2 mice), P8 *n* = 8 (4 mice), P9 = 2 (2 mice), P13 *n* = 10 (6 mice), P16 *n* = 17 (12 mice) and P21 *n* = 14 (13 mice). **D**–**F** Dot plots showing the mean +/− SD of **D** the segment number, **E** segment length, and **F** the total LYVE1-positive vessel length. Number of analyzed ventral ear pinna for P4 *n* = 16 (in 8 mice), P6 *n* = 8 (5 mice), P8 *n* = 4 (4 mice), P13 *n* = 6 (5 mice), P16 *n* = 6 (6 mice), and P21 *n* = 6 (6 mice). Source data for Fig. 1C–F are provided as a Source data file.

To characterize the postnatal morphogenetic process of the dermal lymphatic capillary network, we analyzed mouse ear pinna size and ventral lymphatic capillary network structure throughout its postnatal development and upon maturity at P21 (Supplementary Fig. 1A-C). Mouse ear pinna consists of cartilage and adipocyte layer-separated ventral and dorsal halves. In the first postnatal days, pre-existing LV network is located in the deep dorsal dermis, whereas the ventral side is devoid of LVs (Supplementary Movie 1). At P4, several trees of LYVE1+ LVs invade the ventral dermis from the ear pinna stalk and grow towards the tip of the ear pinna (Fig. 1A). At P6, the pre-existing deep dorsal LV network sprouts to the ventral dermis at few locations close to the ear pinna tip, and expands horizontally on the superficial dermal layers to form large tree-like structures, which are linked to deep dorsal LV network (Fig. 1A, B, Supplementary Fig. 1D–F, and Supplementary Movies 2 and 3). Some of the ventral tip-trees did not show an obvious connection to the deep dorsal network, which may be caused by weaker staining deeper in the tissues or, possibly, a de novo origin from lymphatic progenitors^25–28^. The deep dorsal network sprouted also to superficial dorsal layers on multiple locations to form smaller sub-trees (Supplementary Movie 4). Between P8 and P13, the trees growing from the tip and stalk have occupied the superficial layer of the whole ventral dermis, while both the ear pinna and the LV network continue to grow until P21 (Fig. 1A–F and Supplementary Movies 5 and 6). The expansion of the lymphatic capillary network is accompanied by the differentiation of lymphatic capillaries and collectors^24^. On the ventral side, at P8, 97% of the growing LVs are double positive for LYVE1 and VEGFR3 (Supplementary Fig. 1G, H). Upon maturity, at P21, most of the vessels (91%) have retained LYVE1 and VEGFR3 expression, and thus represent lymphatic capillaries, whereas 9% of vessels are LYVE1- VEGFR3+ lymphatic pre-collectors and collectors (Supplementary Fig. 1G, H and Supplementary Movie 7). As a result of the network expansion and differentiation, the majority of the ventral network, i.e., the capillaries and the pre-collector segments, attains a quasi-2D superficial architecture, whereas rare collector segments are located deeper in the ventral dermis (Supplementary Fig. 1G and Supplementary Movies 7 and 8). The dorsal network, in contrast, consists of two layers, i.e., the abundant deep collector vessels, which are connected to the dorsal superficial lymphatic capillary network (Supplementary Movie 9).

We then wished to understand how the overall growth of the ear pinna and the ventral LV network occurs: This could proceed from the edge of the ear pinna, which would require further tip-branching morphogenesis of a specific population of LVs at the edge, or homogeneously from all points, which would result in an overall dilation of the existing network. First, we reasoned that since hair follicles are created during embryogenesis, an increase in neighboring hair follicle distances is a good proxy for the local postnatal expansion of the skin tissue. Analyses of hair follicle distances of *Sox9-Egfp* mice at P6 and P21 indicated that the ear pinna grows at all locations (Supplementary Fig. 2A–C), which is expected to result in the dilation of the existing network. Second, we performed EdU incorporation assay at P7 and observed uniform lymphatic endothelial cell proliferation both in the tips and the trunk of the LVs (Supplementary Fig. 2D, see also Supplementary Fig. 8H for EdU incorporation at P16). Third, analysis of the network parameters showed a continuous increase in LV segment length throughout the network development (Fig. 1E), consistent with growth-induced expansion of already formed LV branch segments. Altogether, these results support the skin growth-driven global dilation of the established LV network, increasing the spacing of vessels and leading to the elongation of existing LV stalks.

**Fig. 2:**
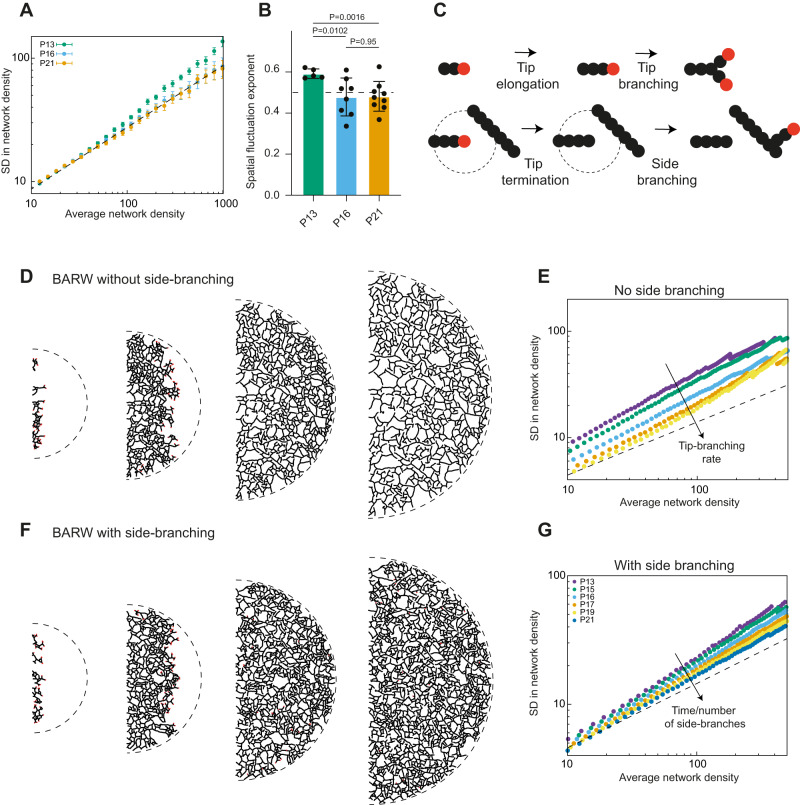
Side-branching optimizes the space-filling by lymphatic vessel network. **A**, **B** Quantification of the efficiency of space-filling of the network as a function of time, measured by the amount of spatial density fluctuations. Fluctuations at later time points (for P16 *n* = 8 ear pinna representing 7 mice and P21 *n* = 9 ear pinna, representing 8 mice) follow the value expected from equilibrium physics (exponent close to $\alpha=0.5$, dashed black line, see Supplementary Information Theory Note for more details), while spatial fluctuations show a larger exponent at P13 ($\alpha=0.60,$ *n* = 5 (ear pinna, representing 5 mice), significantly different from P16, *p* = 0.0102 and P21, *p* = 0.0016, with *p* = 0.95 between P16 and P21). Error bars indicate mean and +/− SD. Two-sided *t* test was used for measuring statistical significance. **C** Sketch of the stochastic rules used in simulations of lymphatic branching morphogenesis via branching and annihilating random walks (BARW). Active growing tips (red) can elongate to give rise to ducts (black), as well as a branch (tip-branching, probability *p*_b_) or terminate their growth if they come too close to neighboring ducts (tip termination/annihilation). We also consider the effect of repulsion on tip growth (see Supplementary Information for details). Finally, we consider the possibility of side-branching (probability *p*_s_), which is the reactivation of growth in a duct. **D**–**F** Exemplary simulations of lymphatic network growth. The size of the ear pinna increases linearly via uniform growth (dilating the existing network) with kinetic parameters inputted from experimental measurements (see Fig. 1C and Supplementary Fig. 2B, C). In the absence of side-branching (**D**, **E**), network growth terminates once tips have reached the edge of the ear pinna, and fluctuations persist. Upon increasing the probability of side-branching (**F**, **G**), active tips are constantly generated. Quantifications of spatial network fluctuations under different model parameters for the tip-branching rate, either without **E** or with **G** side-branching. Without side-branching, we generically observe giant fluctuations due to the inability of the system to correct local density inhomogeneities, while with side-branching, the system can converge to small fluctuations robustly, irrespective of other model parameters (see also Supplementary Movies 10 and 11). Source data for Fig. 2A, B, E, G are provided as a Source data file.

Next, we used the segmented datasets of the LYVE1 + LV networks (Fig. 1 and Supplementary Fig. 2E) to ask how optimal their space-filling properties were over time. We first examined our first time point at which the invading sprouts, from the base of the ear pinna, have reached the border of the ear pinna (P13) and quantified the spatial fluctuations in LV network density^9^. Larger standard deviations are associated with poorer space-filling properties, and can be characterized by the slope (or exponent) of how the standard deviation grows with length scale, or average density (see Supplementary Information Theory Note). More specifically, “giant fluctuations”—a key concept in statistical physics^29,30^—are characterized by slopes larger than 0.5 (1 being the maximal possible value). Thus, we used the standard deviation exponent as a key proxy for how efficient lymphatic tiling is, defining optimal space filling as the absence of large fluctuations. At P13, we found evidence of highly variable space tiling, with typical standard deviation exponents of 0.6 (Fig. 2A, B), close to the ones previously observed in other branched epithelial organs arising from self-organized growth such as the mammary gland^9^. Strikingly, at later time points (P16 and P21), we found that this was resolved, with an absence of large fluctuations (exponents around 0.5, Fig. 2A, B). Similar to the lymphatic capillary network layout, the total LV network, consisting of LYVE1+ and VEGFR3+ capillaries and LYVE1− VEGFR3+ (pre-) collectors, displayed optimal space-filling at P21 (Supplementary Fig. 1I). These results suggest that there could be two complementary processes underlying branching morphogenesis in lymphatic networks: (i) build-up of a non-optimal scaffold via self-organized invasion of space and (ii) space-filling optimization that continues even after the entire dermis has already been covered.

We then turned to biophysical modeling to test different mechanisms of self-organized network growth, that can result in an optimal layout. To model the first phase of lymphatic capillary network growth, i.e., the invasion resulting in the non-optimal coverage of the whole ventral dermis (P4-P13), we asked whether the simplest framework of branching and annihilating random walks—previously used for branched organs, such as mammary gland, pancreas, or branched neuronal growth—could explain the data^9^. This model assumes that branching morphogenesis proceeds from the stochastic invasion of space by elongating and branching tips, which terminate their growth proportional to local lymphatic density (Fig. 2C). We then simulated branched network growth according to these rules, and assumed, based on the data, a two-dimensional hemispherical geometry for the ear pinna. We also took into account the experimentally observed growth of the entire ear pinna (Supplementary Fig. 2A–C and Supplementary Movie 10 and see Supplementary Information Theory Note for details). The resulting in silico networks (Fig. 2D and Supplementary Movie 10) displayed several features of in vivo networks at P13 and, thus, supported the core assumptions of the model: (i) tips terminated in the vicinity of other vessels with little overlap of lymphatic capillaries passing above or below one another, with a characteristic distance between a tip and the closest vessels around it (compare Fig. 2D and insets of Fig. 1A) as posited in a model of tip termination in the vicinity of an existing ductal network (Fig. 2C); (ii) lymphatic networks displayed strong local variability, with branch lengths found to be highly stochastic (characterized by broad distributions with exponential tails), as predicted by a stochastic branching process (Supplementary Fig. 2F); (iii) the resulting networks were characterized by overall constant spatial density, but with large fluctuations, similar to experimental data at P13 (compare Fig. 2A and Fig. 2E). We also found some evidence for self-repulsion of tips as a local guidance mechanism^31^, allowing them to actively avoid ducts, in particular using metrics such as the nematic order parameter (which quantifies the degree of alignment of neighboring branches, see Supplementary Fig. 4A, B and Supplementary Information Theory Note for details).

Next, we sought to explain the near-perfect tiling optimization in the later stages of network construction. We reasoned that two, non-mutually exclusive, processes could account for this: (i) on one hand, network pruning has been shown in a number of systems^32,33^ and could participate in the optimization by removing branches from dense areas. On the other hand, (ii) additional sprouting and sprout elongation could increase the density in less dense areas to correct past inefficiencies in the tiling. We first noted that the overall number of branch segments increases between P13 and P21 (Fig. 1D), supporting that even if both mechanisms are present, side-branching must be more frequent than pruning.

We then systematically tested whether pruning occurs in vivo in dermal lymphatic capillaries. For this, we searched for empty basement membrane sleeves at P21, as an indication of retracted lymphatic capillaries^32^. We did not observe any empty sleeves of single branches/segments (Supplementary Fig. 5), suggesting that large-scale rearrangements are not causative for the optimized network structure. These data, combined with the fact that the overall branch number increased through the phase of tiling optimization from P13-P21, led us to the conclusion that extensive side-branching must occur during this period. This was also consistent with the fact that although branch length increases in time (consistent with global network dilation), it increases less rapidly than the ear radius, which is expected in the presence of side-branching. To theoretically test whether side-branching could participate in making space-filling more efficient, we ran a number of simulations with different assumptions on the parameters of tip-branching invasion with different amounts of side-branching (Fig. 2F, G, Supplementary Movie 11, and Supplementary Fig. 3G–K). We found that side-branching was highly efficient at optimizing space-filling regardless of the initial mechanism of network construction (Fig. 2F, G and Supplementary Fig. 3). Altogether, these modeling approaches and quantitative analyses argued that side-branching could be the key to the process of space-filling optimization in LV networks, something we proceeded to extensively test experimentally.

### Evidence of side-branching via clonal and morphometric analysis

To get more direct insights into the cellular dynamics that drive coverage in lymphatic capillary networks, we conducted a quantitative clonal analysis^34–36^. Random sparse confetti labeling^37^, using the lymphatic endothelium-specific Prox1CreERT2 recombinase^38^, was activated by 4-hydroxy tamoxifen (4-OHT) treatment at P4, 6, 9, or 12, and the ventral ear pinnas were collected at P28. To complement the experimental data, we also mimicked the experiment in silico, by using the same simulations as before and performing computational labeling of cells at four different time points that matched their experimental counterparts.

**Fig. 3:**
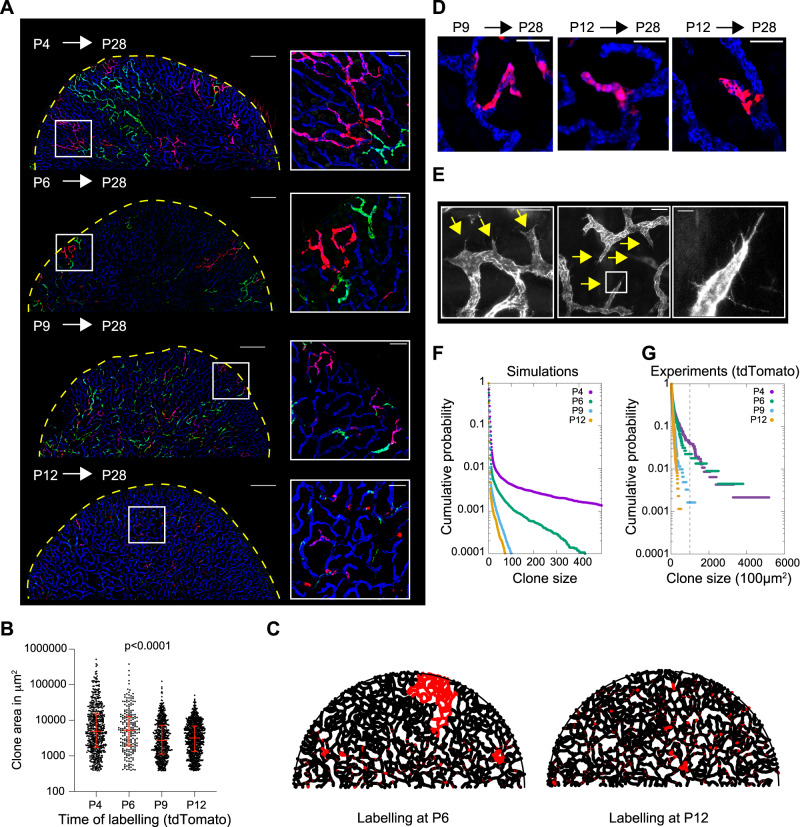
Quantitative lineage-tracing of lymphatic capillary network morphogenesis. **A** Ventral ear pinna dermis of *Prox1CreERT2; R26R-Confetti* mice stained with anti-LYVE1 (blue), anti-GFP (green) and anti-RFP (red). Confetti-mediated genetic labeling of the lymphatic endothelial cells was switched on at the indicated time points and ear pinna were collected at P28. The boxed regions are shown as magnified images. Scale bars are 1 mm in the overview images and 200 μm in magnified images. **B** Quantification of the anti-RFP stained (red) tdTomato clone sizes. Each dot represents a single clone. Median values with interquartile ranges are shown in red. For P4 induced clones *n* = 588 (representing 12 ear pinna in 6 mice), P6 *n* = 224 (7 ear pinna in 4 mice), P9 *n* = 612 (5 ear pinna in 5 mice), and P12 *n* = 868 (10 ear pinna in 5 mice). Kruskal–Wallis test was used for measuring statistical significance (*p* < 0.0001). **C** Exemplary simulations of random clonal labeling (1% of particles irreversibly labeled with red), time-matched to simulate labeling at P6 and P12. **D** Anti-LYVE1 (blue) and anti-RFP (red) stained ventral ear dermis of the *Prox1CreERT2;R26R-Confetti* mice showing uni-clonal branches upon labeling at P9 or P12. The shown images are representative of altogether *n* = 15 ear pinna, representing 10 mice. See Supplementary Fig. 6E for further examples. Scale bars are 100 μm. **E** LYVE1 stained (gray) P13 ventral ear dermis shows sprouts/side branches of existing LVs (yellow arrows). The boxed region is shown as a magnified image. The shown images are representative of *n* = 11 ear pinna, representing 10 mice. Scale bars are 50 μm in the overview and 10 μm in magnified images. **F**, **G** Clone size distribution (cumulative probability) in **F** time-matched simulations and **G** experimental anti-RFP stained tdTomato clones (confetti induction at P4, P6, P9, and P12). Both simulations and experiments show a bimodal clonal behavior upon early clone labeling (with large clones (defined as having an area larger than 10^5^ μm^2^, dashed vertical line) from labeling of active growing tips and smaller clones from inactive ducts) which gradually subsides the later the clones are induced. See Supplementary Fig. 6A–G for additional analyses of the lineage-tracing data set presented here. Source data for Fig. 3B, F, G are provided as a Source data file.

In both simulations and experiments, we found unicolor clones that spanned large regions of the network from the middle parts of the ventral ear pinna to the edges, as well as smaller clones dispersed throughout the network (Fig. 3A–C and Supplementary Fig. 6A–F). The appearance of such large clones, upon labeling during the phase of invasive LV growth, is consistent with our model, in which the LV branching to previously unoccupied regions is driven by a few cells that proliferate near the tips of LVs. Accordingly, large clones were absent from ears in which the recombinase was activated at later time points, when the overall coverage of the ear was already complete (Fig. 3A–C and Supplementary Fig. 6A–D). In these samples of later label induction, there was no correlation between the average clone size and distance from the edge of the ear (Supplementary Fig. 6G). These clones included uni-clonal single branches (Fig. 3D and Supplementary Fig. 6E), which were dispersed throughout the ear and, as expected based on our model of side-branching. There was also clonal expansion in the stalk of the vessels (Supplementary Fig. 6F), which together with uniform EdU incorporation in LV tips and stalks (Supplementary Fig. 2D), supports dermal growth-driven dilation of the LV network.

To further explore the side-branching of existing vessels, we conducted high-resolution imaging at the time of network optimization. Indeed, we observed numerous nascent sprouts in the existing vessels at P13 (Fig. 3E). We then employed two complementary approaches to analyze the relative contributions of the tip-splitting vs. side-branching in driving the LV network expansion. First, manual annotation of nascent branching events indicated that at P6 invasive LV growth into the dermis was characterized by both tip-splitting and side-branching of the growing LV trees, whereas side-branching was increasingly prevalent over time (Supplementary Fig. 7A, B). Second, we analyzed the branching angles of the existing network at different developmental time points: We defined a quantitative measure for the angular configurations around branching points that distinguish lateral branching modes (i.e., side-branching-like) from “fork-like” bifurcations (see Supplementary Information Theory Note for more details) and validated the approach with manually curated side-branching events. This approach allowed us to, retrospectively, infer the history of the established branches. The results were consistent with an increasing fraction of side-branching events as a function of time (Supplementary Fig. 7C–E). Altogether, these lineage tracing and branching analyses at key time points of network development show that side-branching is present in the developing LV networks.

To get a more quantitative insight into the growth of the network, we first plotted clone size distribution across many simulations. We found that at early time points, it consistently displays two tails/populations (as visualized in a semi-log plot, Fig. 3F), which is in sharp contrast to the later time point, showing a single tail/population. This qualitative difference is underlined by the fact that early and late distributions have very different shapes even after normalizing for average clone size (Supplementary Fig. 6C), providing evidence against a simple global decrease of proliferation in a single population^39^. The first exponential tail with a very small average clone size arises due to the stochastic proliferation of non-tip cells (required to extend the vessel stalk to keep up with the ear pinna growth). The second tail, characterized with very large but still highly variable clone sizes (defined as being larger than 10^5^ μm^2^), represents the stochastic branching dynamics of the tip cell, according to the branching and annihilating random walk framework, where all tip cells compete neutrally for space via branching and growth. The average size of this second population decayed rapidly as a function of the time of labeling, presumably because there was less space left to be covered at later time points (Fig. 3F). These findings were robust to different initial conditions of the simulations, for instance, the number and location of initial tips/LV trees starting the branching morphogenesis at P4 (Supplementary Fig. 6H and Supplementary Movie 12, see Supplementary Information Theory Note for more details). When we compared these predicted features to the experimental distributions, plotted in the same manner (compare Fig. 3F to Fig. 3G), we found good agreement: in vivo (i) the distribution of small clones followed an exponential curve at all time points, as expected based on the local stochastic proliferation patterns, (ii) at early time points, a small second population (less than 5% of all clones) was orders of magnitude larger, and displayed extreme variability (long tails in the distributions) and (iii) we found a sharp decay in the size of this second population as a function of time (Fig. 3G). Altogether, the clonal analyses across developmental time points qualitatively and quantitatively validate our model of lymphatic network branching morphogenesis, including both tip-driven global self-organization and local space filling by side-branching.

### Genetic manipulation of side-branching

**Fig. 4:**
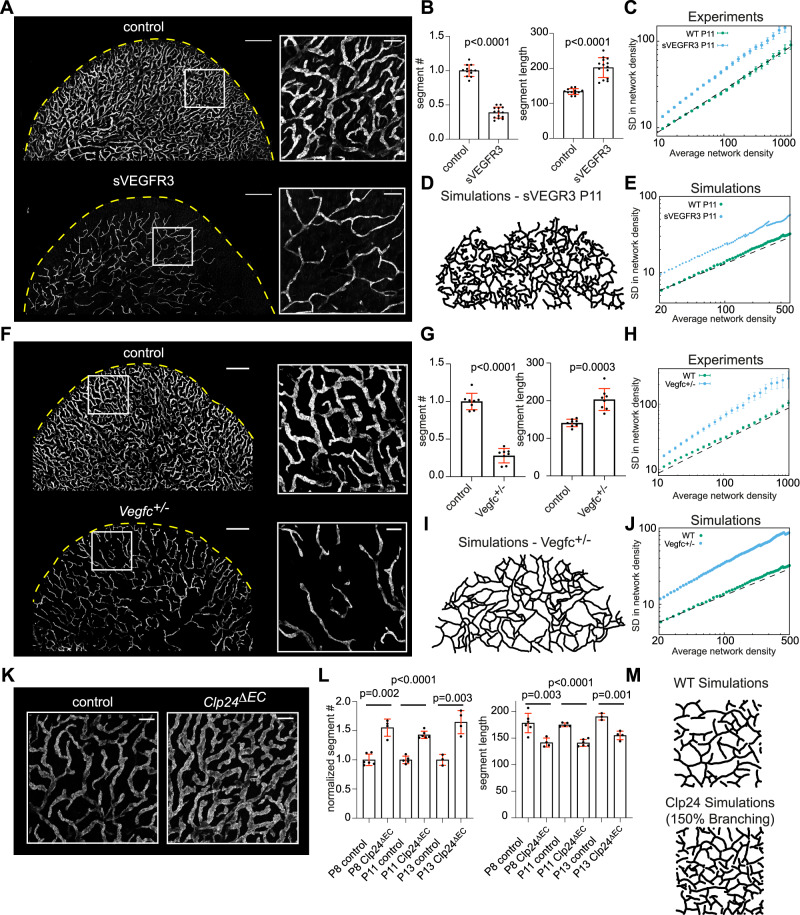
Perturbations of VEGF-C to VEGFR3 signaling influence the side-branching and space-filling efficiency of lymphatic capillary networks. **A**–**C** LYVE1 stained ventral ear pinna dermis of control (*n* = 12 ear pinna, representing 6 mice) or soluble VEGFR3 receptor (VEGF-C ligand trap) (*n* = 14 ear pinna, representing 7 mice) treated (from P11 to P21) mice. The dashed line indicates the ear pinna boundary. The boxed regions are shown as magnified images. **B** Mean +/− SD of the segment number (*p* < 0.0001), normalized to the average of controls (set as 1), and median segment length (*p* < 0.0001) of control (*n* = 12 ear pinna) and sVEGFR3 (*n* = 14 ear pinna) treated mouse ear pinna ventral dermis lymphatic capillaries. **C** Quantification of the spatial fluctuations at P21 in control (*n* = 12 ear pinna) or sVEGFR3 (*n* = 14 ear pinna) treated mice, showing enhanced fluctuations in the latter (slope $\alpha=0.56$). **D**, **E** consistent with simulations with abolished branching from P11 (as well as random pruning, see Supplementary Information Theory Note for details). **F**–**H** LYVE1-stained ventral ear dermis of control (*n* = 8 ear pinna, representing 4 mice) and *Vegfc*^*+/−*^ mice (*n* = 8 ear pinna, representing 5 mice). The yellow dashed line indicates the ear pinna boundary. The boxed regions are shown as magnified images. **G** Mean +/− SD of segment number (*p* < 0.0001), normalized to the average of controls (set as 1) as in (**B**), and median segment length (*p* = 0.0003) of lymphatic capillaries in control (*n* = 8 ear pinna) and *Vegfc*^*+/−*^ (*n* = 8 ear pinna) mice. **H** Quantification of the spatial fluctuations at P21 in wild-type (*n* = 8 ear pinna) and *Vegfc*^*+/−*^ (*n* = 8 ear pinna) mice, showing enhanced fluctuations in the latter (slope $\alpha=0.65$), **I**, **J** consistent with simulations with abolished side-branching and decreased tip-branching (to 25% of its WT value, based on (**G**). **K** LYVE1-stained ventral ear dermis of control (*n* = 6 ear pinna, representing 4 mice) and *Clp24*^*ΔEC*^ mice (*n* = 4 ear pinna, representing 2 mice). *Clp24*^*ΔEC*^ was deleted at P8. See Supplemental Fig. 9A for further examples. **L** Mean +/− SD of the segment number, normalized to the average of controls (set as 1), and median segment length of control and *Clp24*^*ΔEC*^ mouse ear pinna ventral dermis lymphatic capillaries upon 4-OHT mediated *Clp24* deletion at p8 (control n = 6 ear pinna, representing 4 mice and *Clp24*^*ΔEC*^ *n* = 4 ear pinna, representing 2 mice), P11 (control *n* = 5 and *Clp24*^*ΔEC*^ *n* = 6 ear pinna, representing 3 mice each) or P13 (control n = 3 and *Clp24*^*ΔEC*^ *n* = 4 ear pinna, representing 3 mice each). For comparison of segment number in controls and *Clp24*^*ΔEC*^, *p* = 0.002 (Clp24 deletion at P8), *p* < 0.0001 (at P11), and *p* = 0.003 at (P13), whereas for comparison of segment length *p* = 0.003, *p* < 0.0001, and *p* = 0.001, respectively. **M** Close-up of simulations for WT and *Clp24*^*ΔEC*^ mouse (150% branching rate compared to WT), showing good qualitative agreement with the data. Scale bars for overview and magnified images in (**A**) and (**F**) are 1 mm and 200 μm, respectively, and for (**K**) 200 μm. In (**B**), (**G**), and (**L**), two-sided Welch’s *t* test was used for measuring statistical significance. Source data for Fig. 4B, C, G, H, L are provided as a Source data file.

To experimentally test if side-branching is essential for tissue coverage optimization by lymphatic capillaries, we sought to functionally interfere with it. Given that VEGF-C binding to VEGFR3 is essential for the formation of lymphatic endothelial tip cells in lymphangiogenesis^40,41^, we sequestered VEGF-C by adeno-associated virus (AAV) vector-mediated expression of a VEGF-C/D ligand trap starting at P11, i.e., after overall ear pinna coverage was achieved but prior to the phase of network optimization^42^. Indeed, at P13 the ligand trap resulted in a severe reduction in the number of nascent side branches, emphasizing the necessity of VEGF-C for sprouting of existing vessels (Supplementary Fig. 8A, C). Also, at P13 and P16, tip cell arrow-like morphology was largely lost and LV tips appeared blunted, suggesting a loss of an invasive phenotype (Supplementary Fig. 8B–E). At P21, In comparison to control AAV-treated mice, the expression of the ligand trap resulted in sparse lymphatic capillary networks with fewer branches, which were longer on average (Fig. 4A, B), consistent with a strong inhibition of side-branching/sprouting. The remaining lymphatic network was composed of thin vessels that failed to reach the expanding ear edge (Fig. 4A). This finding was consistent with a drastic decrease of cell proliferation as evidenced by a decrease in EdU incorporation into lymphatic endothelial cell DNA (Supplementary Fig. 8F–H). Importantly, analysis of ear pinna dermis space-filling by LVs indicated increased variance and larger spatial fluctuations in ligand-trap treated mice (Fig. 4C). Numerical simulations with abolished branching after P11, gave results that were consistent with the in vivo experiments (Fig. 4D, E). Since sequestration of VEGF-C/D was also associated with rare occasions of empty basement membrane sleeves (Supplementary Fig. 8I), a likely marker of vessel regression^32^, we also modeled the possible effect of local pruning. However, different levels of pruning did not significantly change space-filling efficiency (see Supplementary Fig. 4C and Supplementary Information Theory Note for more detailed discussion and modeling), supporting a central role of side-branching in network optimization.

To test the role of side-branching further, we examined *Vegfc*^*+/−*^ haploinsufficient mice (Fig. 4F). Whereas homozygote *Vegfc* deletion is embryonically lethal, *Vegfc* heterozygote mice are viable, show reduced VEGF-C protein levels and display lymphatic capillary hypoplasia and reduced tissue fluid draining capacity^43^. V*egfc* heterozygosity resulted in networks that could tile the overall ear (Fig. 4F), indicating that invasive tip-driven morphogenesis can still proceed, while increased branch length suggested strongly impaired side-branching. Importantly, the overall density of branches was decreased and the density fluctuations were increased with a fluctuation exponent of *α* = 0.65 at P21 comparable to values observed at P13 for wild-type (Fig. 4F–H). This was consistent with our model with defective LV side-branching (Fig. 4I, J).

Finally, to test the reverse effect of increased VEGF-C signaling on the lymphatic capillary network, we deleted the claudin-like protein 24 (*Clp24*) gene^44^. Constitutive deletion of *Clp24* was reported to result in increased VEGFR3 signaling and enlargement of dermal lymphatic capillaries^45^. We induced deletion of *Clp24* in endothelial cells by 4-hydroxy tamoxifen injection into *VE-cadherin-CRE-ERT2; Clp24*^*lox/lox*^ mice (*Clp24*^*ΔEC*^) at P8, P11 or P13, i.e., at a time when the ventral ear dermis is already mostly covered by non-optimal network scaffold, and analyzed the mature P21 networks. Gene deletion at the three time points resulted in the enlargement of lymphatic capillaries when analyzed at P21 (Fig. 4K and Supplementary Fig. 9), confirming the endothelial specificity of the constitutive phenotype. In comparison to 4-hydroxy tamoxifen-treated *Clp24*^*lox/lox*^ control mice, the total number of segments increased whereas segment length decreased, indicating increased side-branching (Fig. 4L). Density fluctuation analyses indicated that the *Clp24*^*ΔEC*^ LV networks uniformly covered the dermis, as in the control mice (Fig. 4M and Supplementary Fig. 9B), indicating an active control mechanism even in the presence of hyper sprouting. In conclusion, although VEGF-C controls multiple aspects of lymphatic endothelial biology, including tip cell phenotype^40,41,46^, our results show that high levels of VEGF-C to VEGFR3 signaling are required for frequent side-branching and, thus, optimal tiling.

### Nascent side branches target low-density regions

**Fig. 5:**
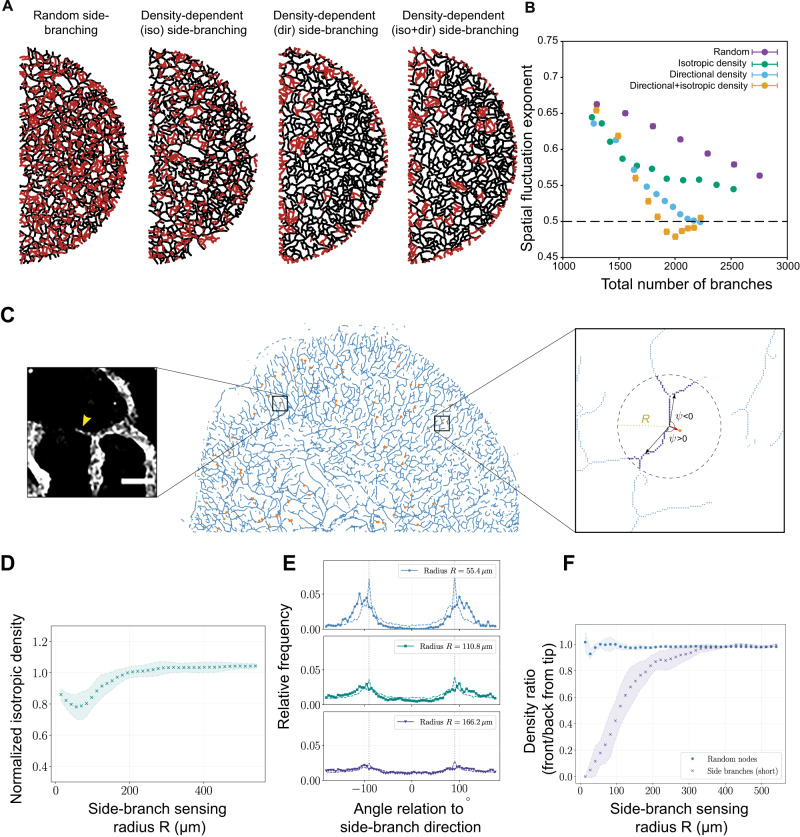
Side-branching targets low-density regions to ensure parsimonious space-filling. **A** Exemplary simulations under different assumptions for side-branching (red): random side-branching (each vessel has an equal probability to re-activate tips), density-dependent isotropic (iso) side-branching (which occurs preferentially in regions of overall low density), directional (dir) side-branching (which occurs in directions of relative low density), and combinations of directional and isotropic sensing. **B** Space-filling efficiency in simulations quantified by spatial fluctuation exponent as a function of the total number of branches in the network. We find that both density-sensing mechanisms allow for more efficient space-filling with a smaller overall number of branches, with an additive effect when combined (orange dots). **C** Representative skeletonized lymphatic network (blue) from an LYVE1-stained P13 ventral mouse ear pinna (*n* = 3 ear pinna, representing 3 mice). Orange nodes represent manually curated nascent sprouts. Boxed region (left): Magnified original image of a nascent sprout (yellow arrowhead) (scale bar: 50 μm). Boxed region (right): The initial directionality of a side branch can be represented by a vector (red arrow) connecting the root of the side branch to the side branch terminal tip. Neighboring branch segments (purple) within a circle of radius *R* (dashed line) can be used to determine their angle to side branch ψ. **D** Ratio of isotropic densities around side branches *ρ*_*s*_ to densities *ρ*_*r*_ around random points on the network for different values of R. Density ratios smaller than 1 for *R* < 200 μm indicate that side branches initiate in regions of smaller isotropic densities of LVs compared with randomly selected regions of the network. For larger *R*, both densities converge to the same value. **E** Relative frequencies (solid lines with markers) of angles to side branch ψ for different values of *R*. Dashed lines represent distributions corresponding to randomly selected points on the branched network. Dotted vertical lines represent ψ = ±90°. **F** Ratio of probabilities to find LVs with an angle to side branch of |ψ| < 45° and |ψ| > 135°, i.e., neighbors that lie in the “front” and “back” of the side branch, for different values of *R*. Probability ratios around side branches (purple crosses) indicate that side branches initiate preferably into regions of lower density (ratio smaller than 1), in contrast with densities around random regions (blue circular markers) that exhibit an unbiased front/back ratio equal to 1. Metrics in (**D**–**F**) are calculated over *n* = 135 manually labeled nascent sprouts, representing three P13 ear pinna and mice. Plot markers and shaded error bands in (**D**) and (**F**) indicate mean values and +/−SDs. Source data for Fig. 5B, D–F are provided as a Source data file.

Having tested experimentally the role of side-branching in optimal space-filling by lymphatic networks, we then explored whether the amount of side-branching that can be inferred from the data was quantitatively enough to decrease LV density fluctuations between P13 and P16-21. So far, we have modeled “random” side-branching events which can occur at any location in the network. Although this hypothesis has little consequence on the resulting clone size distributions discussed above, we reasoned that it could be a wasteful way to optimize coverage, as this also creates numerous side branches in already dense regions (Fig. 5A). We thus tested computationally alternative strategies of optimization, in which nascent sprouts sense local LVs. This could be driven, for instance, by (i) increased side-branching probability in regions of low overall density (isotropic sensing) or (ii) active directed growth towards the low-density regions (directional sensing)^47^. We found that implementing these two mechanisms of density sensing drastically decreased the time and/or frequency of side-branching necessary to decrease the LV density fluctuation exponent (Supplementary Software 1). In other words, such density-dependent mechanisms would rapidly and parsimoniously optimize space-filling (see Fig. 5A, B) compared to random side-branching.

We thus went back to our segmented dataset at P13 (Fig. 1 and Supplementary Fig. 2E), where numerous nascent sprouts were observed (Fig. 3E), to see if we can find signatures of such regulations in vivo. We first manually located the nascent sprouts, which had a slender or arrowhead-like phenotype, and mapped the local positions of nascent sprouts on the entire segmented network (Fig. 5C). To test the density-dependent regulation of side-branching, we then calculated (i) the isotropic branch density around nascent sprouts, and compared it to random points in the network, and (ii) the relative branch densities in the direction of a nascent sprout, compared to the opposite direction (as illustrated in Fig. 5C). We found that up to the length scale of $R\approx 150\mu m$, isotropic densities around nascent sprouts were indeed smaller than in the rest of the network, indicating that side-branching occurred preferentially in low-density regions (Fig. 5D and Supplementary Fig. 10, see Supplementary Information Theory Note for details). Surprisingly, not only the frequency of nascent sprouts but also their growth directionality was affected by density. The side branches grew specifically in directions of low pre-existing LV density (again up to length scales of around $R \, \approx \, 150 \, {{{{{\mathrm{\mu}}}}}}{{{{{\rm{m}}}}}}$, Fig. 5E, F and Supplementary Fig. 10). Overall, our analysis of the local network density around the nascent side branches, thus, strongly suggests that the new sprouts actively sense the local density of neighboring lymphatic capillaries to contribute to a rapid optimization of space tiling at P13.

## Discussion

Lymphatic capillary density is a key parameter determining the efficiency of the tissue fluid drainage and also the trafficking of the dendritic cells via the LVs. Here, we show how the branching morphogenesis of lymphatic capillaries can result in optimal coverage of mouse dermal tissue in a self-organized manner, which can be quantitatively modeled by using a minimal set of local rules for branching. We present a two-tier model for branching, where the first phase of stochastic branching, elongation and termination of growth generates an LV scaffold covering the entire ventral ear dermis in a non-optimal manner. Subsequently, side-branching from existing vessels of the scaffold allows for efficient optimization of branched network coverage, as well as its overall growth in response to uniform expansion of the surrounding tissues.^48^ Furthermore, we find experimentally that side-branching of LV sprouts actively targets low-density regions of the network, which enables optimal coverage in a highly parsimonious and economical manner. Such targeted growth presents a strategy that deviates from previously proposed mechanisms, which rely on network remodeling and pruning. For instance, the first phase of pancreatic development has been proposed to rely on a transition from a connected plexus of ducts towards a simple branched tree which optimizes viscous dissipation^33^. Development of the blood vessel network also involves pruning, in which segments with blood flow (i.e., functional parts of the networks) are preserved, whereas segments devoid of blood flow regress^32^, presenting a process that allows efficient optimization of transport in biological networks^10^. In the case of lymphatic capillaries, lymph flow at 1–30 μm/s^49,50^ is considerably weaker than flow in blood capillaries, thus the possible differences in the flow might be insufficient for distinguishing functional units from non-functional ones. In the absence of pruning, an excessive number of segments might also lead to inefficient lymph flow and slow down dendritic cell pathfinding on their way to lymph nodes^51^. Thus, guided/directional growth may be a superior strategy in the absence of a clear distinction between functional vs. non-functional network segments. Interestingly, it was recently shown that LVs do not obey Murray’s Law^52^, which posits a scaling between the widths of mother and daughter vessels based on the minimization of flow resistance in vascular systems. However, optimizing space-filling by targeted growth into low-density regions might be an alternative strategy of lymphatic vasculature to ensure efficient tissue fluid and immune cell transport.

The ability of sprouts to sense the proximity of existing lymphatic capillaries could rely on at least two non-mutually exclusive mechanisms: (i) The VEGF-C growth factor is essential for lymphangiogenesis and its cognate receptor VEGFR3 is expressed on the surface of all lymphatic endothelial cells, while VEGFR3 in the tip cells is crucial for the migration/tissue invasion of the sprouts^40,41^. Given that VEGF-C binding to VEGFR3 promotes endocytosis and thus clearance of VEGF-C from extracellular space^53^, VEGFR3 may act as a sink, decreasing VEGF-C in high LV density regions^54^. In line with this hypothesis, mosaic deletion of VEGFR3 in lymphatic endothelium resulted in hyper sprouting of the remaining wild-type lymphatic endothelial cells in VEGF-C-dependent manner^40^. Also, TIE1 deletion-induced decrease in lymphatic endothelial cell surface VEGFR3 levels resulted in chaotic LV networks^55^, as if LVs were unable to sense the local LV density. Thus, VEGFR3 may play a dual role: on the one hand, it would act as a sink, causing reduced levels of VEGF-C in the LV dense areas and, on the other hand, it drives sprout formation in the high VEGF-C areas. (ii) Alternatively, lymphatic capillaries could express repellents that deflect or regress sprouts. For example, semaphorin-plexin signaling between blood vessel and LVs has been shown to induce deflection of LVs during development^56^. In both possible modes of density sensing, lymphatic endothelial sprouts should be able to reach out to avoid the local suppression by the “mother vessel.” One could envisage a scenario, where sampling of the environment with long filopodia-like protrusion could guide decision-making before sprout formation, as in the case of angiogenesis^57^, or where nascent sprouts could form in excess and then become stabilized only at low-density regions. Adapting live-imaging methods to lymphatic network morphogenesis would be useful in the future to discriminate between these different possibilities, as well as to understand more quantitatively the dynamics of the transition between tip and side-branching during development.

Recent studies have shown that lymphvasculogenesis of lymphatic endothelial progenitors contributes to, at least, part of dermal, mesenterial, and heart LV networks^25–28^. Islets of lymphatic progenitor expand and fuse together, followed by sprouting lymphangiogenesis of the initial scaffold^25–27^. Thus, the major difference is that in lymphvasculogenesis lymphatic segments grow towards and fuse with each other, whereas in sprouting lymphangiogenesis, as discovered here, locally dense LV networks suppress sprouting/growth. Further work is required to reveal the molecular switch that alters the effect of neighboring LVs from attraction (fusion of vessel segments) to suppression (as identified here).

Here, we used simple quasi-2D LV networks of ventral mouse ear pinna as a model of lymphatic branching morphogenesis. Our results on the self-organization and optimal tiling of lymphatic capillary networks illustrate the power of simple local rules that may guide biophysical interactions between neighboring regions of the LV network and how they give rise to robust developmental structures in vivo. We expect our findings to have direct implications in other contexts of sprouting lymphangiogenesis, such as thicker dermis or intestine^58–60^, where a combination of LV density sensing and localized production of LV guidance cues, including VEGF-C, may steer the growth of the final sprouts. The identification of the signals that mediate lymphatic endothelial self-sensing would be important for the induction of regenerative lymphangiogenesis and expansion of mature lymphatic capillary networks to facilitate tissue clearance and adaptive immunity in tumorigenesis and inflammation.

## Methods

### Mice

Adult wild-type C57BL/6Rcc mice were from Envigo, whereas the P1-P21 C57BL/6Rcc mice were produced at the local animal facility. *Clp24*^*flox/flox*^ mouse strain was crossed to *Cdh5(BAC)-CreER*^*T2*^ strain and maintained in C57BL6 background^45,61^. *Vegfc*^*+/−*^ mice were in ICR (CD-1) background^43^. *R26R-Confetti*^*flox*^ mice (JAX 013731) were crossed to *Prox1-CreER*^*T2*^ mice in mixed background^37,38^. Transgenic *Sox9-Egfp* mice were in B6/Crl background (an albino mutant of C57BL/6 strain)^62^. Ear pinna of both female and male mice were used for analyzes. All the mice were bred and handled according to the local ethical regulations. Animal housing was in conditions of 12-h light/dark cycle, 23 °C ambient temperature and approximately 50–60% humidity. Experimental procedures were approved by the Project Authorization Board in Finland (animal license ESAVI/30523/2019 and ESAVI/40857/2022).

For conditional tamoxifen-induced gene deletions, mice were treated with 4-hydroxy tamoxifen (4OHT; Sigma H6278) as follows. *Cdh5(BAC)-CreER*^*T2*^*;CLP24*^*lox/lox*^ mice received daily intraperitoneal injections of 30 μl of ethanol dissolved (25 mg/ml) and then olive oil diluted (1:7.5, final concentration 3.3 mg/ml) 4-OHT. The pups were treated daily for three days from P8 to P10, from P11 to P13, or from P13 to P15. Ear pinna were collected for analysis at P28.

Clonal labeling in *Prox1-CreER*^*T2*^*;Confetti*^*wt/flox*^ mice was induced by a single 4-OHT treatment. For induction of labeling at P4 the mice were treated with an intragastric injection of 2.5 μl ethanol dissolved 4-OHT (25 mg/ml). For labeling at P6, P9 or P12, the mice were treated with an intraperitoneal injection of 25 μl of ethanol dissolved and olive oil diluted 4-OHT (final concentration of 0.63 mg/ml). The initial whole-mount microscopy showed dim or no signal and thus we stained the expressed labels with anti-RFP and anti-GFP antibodies to be able to more accurately reconstruct clones quantitatively across the entire ear (see below). Anti-RFP stains only the tdTomato clones (see quantification in Fig. 3B, G and Supplementary Fig. 6A), whereas anti-GFP stains GFP, YFP, and mCerulean expressing clones (see quantification in Supplementary Fig. 6A, B, D). The used low dosage of 4-OHT allowed sparse labeling and thus identification of independent clones.

### VEGF-C ligand trap treatment

Wild-type mice were intraperitoneally injected at P11 with adeno-associated virus (AAV; 1.5 × 10^11^ virus particles) encoding for the soluble extracellular ligand-binding part of the VEGF-C receptor VEGFR3 (exons 1–4) or, as a control, non-ligand-binding part (exons 4-7) of VEGFR3^42,63^. Mice were culled and ears were collected at P13, P16, or P21.

### Ear pinna preparation

The mice were culled and the ear pinna were prepared for whole-mount immunofluorescence staining as follows: The ear pinna of P1-P9 mice were cut off from the skin of the skull and incubated in 20 mM EDTA at +37 °C for 45–60 min, after which the epidermis was peeled off and the ear pinna were fixed in 4% PFA at RT for 25’. The ear pinna of P13 mice were separated from the skin of the skull by cutting and the whole ear pinna were fixed overnight at +4 °C in 4% PFA, followed by separation of ventral and dorsal ear pinna. For the ear pinna of P16 and P21 mice, the ventral and dorsal ea pinna sheets were, first, separated by forceps and, then, fixed in 4% PFA at RT for 25’. After fixation, the ear pinna were washed three times with PBS.

### Ear pinna wholemount staining

The ears were blocked with 3% bovine serum albumin and incubated with primary antibodies one to two days at +4 °C on the rocker. The ear pinna were washed three times with PBS and then incubated with secondary antibodies one to two days at +4 °C or for 2 h at RT, after which ear pinna were washed three times with PBS. After the staining, the ear pinna of the P1–P13 mice were cleared with BABB as follows: after three washes in methanol, the ear pinna were further incubated for 30–60’ in methanol. Then the ear pinna were washed four times with BABB and then incubated for a further 1–2 h in BABB. The cleared ear pinna were mounted in BABB in between two coverslips.

The used primary antibodies were: rat anti-mouse LYVE1 (1:300; R&D systems MAB2125), goat anti-VEGFR3 (1:150; R&D AF743), rabbit anti-collagen IV (1:150; Abcam Ab6586), chicken anti-GFP (1:1000; Abcam ab13970) and rabbit anti-RFP (1:500; Rockland 600-401-379). The used secondary antibodies were: Alexa Fluor 488 donkey anti-chicken (1:400; Jackson laboratories 703-545-155), Alexa Fluor 594 donkey anti-rat (1:400; Invitrogen A21209), Alexa Fluor 594 donkey anti-rabbit (1:400; Invitrogen A21207), Alexa Fluor 594 donkey anti-goat (1:400; Invitrogen A11058), Alexa Fluor 647 donkey anti-rat (1:400; 712-606-153), Alexa Fluor 647 donkey anti-goat (1:400; Invitrogen A21207).

The mounted stained ear pinna were imaged with (i) Zeis LSM780 or (ii) 880 inverted confocal microscopes equipped with Zeis ZEN 2010 or Zeis ZEN2 software, respectively, and the following objectives ×10 Plan-Apochromat 0.45, ×20 Plan Apochromat 0.80 and ×63 Plan Apochromat 1.40, or (iii) Andor dragonfly spinning disc microscope equipped with Fusion 2.0 software and ×20 Plan Apochromat 0.74 objective. FiJi image analyses software was used to set the brightness and contrast of the images.

### EdU labeling

The P7 wild-type mice were treated with EdU (50 mg/kg) for 4 h (Supplementary Fig. 2D), whereas the P15 control and sVEGFR3 expressing mice were treated for 20 h (collected at P16; Supplementary Fig. 8F–H). The ear pinna were collected and prepared as described above. The EdU was labeled with Click-it technology according to the protocol provided by the manufacturer (Invitrogen C10339) followed by LYVE1 staining.

### Ear pinna LV network analyses

For the network analyses, we used either full ventral ear pinna images (Figs. 1, 3, and 5 and Supplementary Figs. 2B, C, 6, 7, 8A, B, and 10) or a cropped area in the central part of the ear pinna (Figs. 2A, B and 4B, C, G, H, L and Supplementary Figs. 1H–J, 2F, 4A, 8D, F–H, and 9). The original images were segmented with ilastik^64^ and the network parameters were analyzed with Imaris image analyses software (Bitplane; Supplementary Fig. 1A, C–F and Fig. 4B, G, L), or the segmented images were skeletonized (Supplementary Fig. 2E) for analyses of density fluctuations (Figs. 2A, B and 4C, H and Supplementary Figs. 1I and 9B), LV network density at the site of nascent sprouts (Fig. 5 and Supplementary Fig. 10), distribution of P13 branch lengths (Supplementary Fig. 2F) and nematic alignment of LVs (Supplementary Fig. 4A) (see Supplementary Information Theory Note for details). Since the imaged ear pinna contains small areas of poor staining (due to tissue preparation), the results of analyzed area (i.e., segment number and total network length), were normalized to total ear pinna area (analysis shown in Fig. 1D, F). For the measurement of lymphatic capillary vs. total LV network length (Supplementary Fig. 1H), LYVE1 staining was segmented with ilastik and skeletonized. The segmented binary images were used to mask the corresponding original VEGFR3 image and the skeletons of the LYVE1- VEGFR3+ vessel segments were drawn manually. The skeletons were used to quantify the length of lymphatic capillaries (LYVE1+VEGFR3+) and the total length LVs (capillaries and collectors). For the clonal analyses, we used Imaris to segment and analyze the clones residing in LYVE1 positive lymphatic capillaries (Fig. 3B and Supplementary Fig. 6A, B).

For the classification of branching events (Supplementary Fig. 7A, B), we manually annotated nascent arrow-head-like branches (20-200 μm in length): tip-splitting events displayed a splitting of a tip to two slender branches, which were approximately of similar width, whereas side-branching events were more distant to the tip of the existing vessel and the sprout was considerable of smaller width than the existing vessel.

To visualize the expanding ventral LV networks at P6 and P8 (Fig. 1B and Supplementary Fig. 1F), we manually drew the skeletons on top of the original confocal stack by using FiJi image analysis software. For clarity, only the drawn trees are shown, and not the original stack. The FiJi color-code tool was used to highlight the connection between the ventral and dorsal networks (Supplementary Fig. 1E).

The manuscript images have been prepared first by using FiJi (NIH) and Imaris (Bitplane) image analysis software, followed by Adobe Photoshop and Adobe Illustrator software.

### Ear hair follicle analyses

The distances of hair follicles in P6 and P21 ears were analyzed with Imaris. After a distance transformation (inward from the ear pinna edge), the sites of hair follicles were marked and the distance of the hair follicles to the ear border and closest hair follicle were analyzed.

### Statistical analyses

Measurement were taken from distinct samples and all the datapoints represent independent samples. We used Prism software (GraphPad) to test the normality of the data, after which parametric T-test with 2-tailed distribution and Welsch correction were used for testing the statistical significance, except for Fig. 3B and Supplementary Fig. 6A, B for which non-parametric Kruskal–Wallis test was used. Error bars and bands show the standard deviation (SD).

### Reporting summary

Further information on research design is available in the Nature Portfolio Reporting Summary linked to this article.

### Supplementary information

Supplementary Information

Description of Additional Supplementary Files

Supplementary Movie 1

Supplementary Movie 2

Supplementary Movie 3

Supplementary Movie 4

Supplementary Movie 5

Supplementary Movie 6

Supplementary Movie 7

Supplementary Movie 8

Supplementary Movie 9

Supplementary Movie 10

Supplementary Movie 11

Supplementary Movie 12

Supplementary Software 1

Reporting Summary

### Source data

Source Data

## Data Availability

All data supporting the findings of this study are available from the corresponding author upon request. Source data are provided with this paper.

## Code availability

Source code and typical outputs of branching simulations are provided with this paper.
