# Supplementary material for: Self-organized and directed branching results in optimal coverage in developing dermal lymphatic networks
Source: Nat Commun. 2023 Sep 21;14:5878. doi: 10.1038/s41467-023-41456-7 (PMC10514270; doi:10.1038/s41467-023-41456-7)
Supplement: Supplementary file 1 — Supplementary Information [file 41467_2023_41456_MOESM1_ESM.pdf]

Supplementary Information

Self-organized and directed branching results in optimal coverage in developing dermal lymphatic networks

Mehmet Can Ucar[1,*], Edouard Hannezo[1,*#], Emmi Tiilikainen[2], Inam Liaqat[2], Emma Jakobsson[2], Harri Nurmi[2,3], Kari Vaahtomeri[2,3,#]

[1] Institute of Science and Technology Austria (IST Austria), Am Campus 1, 3400 Klosterneuburg, Austria

[2] Translational Cancer Medicine Research Program, University of Helsinki, Biomedicum Helsinki, Haartmaninkatu 8, 00290 Helsinki, Finland

[3] Wihuri Research Institute, Biomedicum Helsinki, Haartmaninkatu 8, 00290 Helsinki, Finland

*Shared first author

#Correspondance

edouard.hannezo@ist.ac.at

kari.vaahtomeri@helsinki.fi

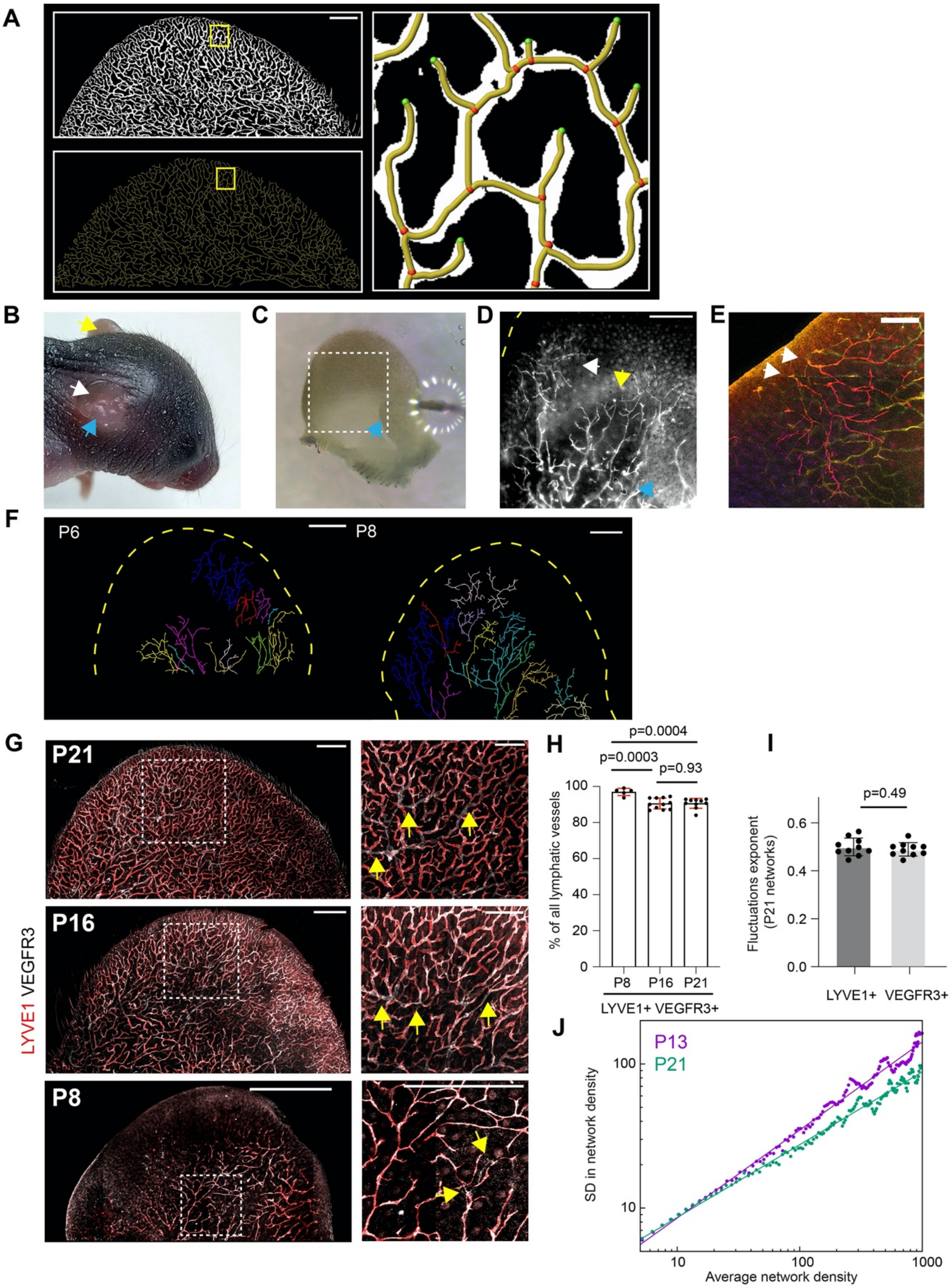

Supplementary Figure 1: Analysis of the lymphatic vessel network properties

Representative overview images of segmented wild-type P21 mouse ear pinna dermal LV-network (white), the corresponding IMARIS skeleton of the network (yellow) and an overlay

as a magnified image (n=6 ear pinna, representing 6 mice). Branch points are indicated with orange nodes and tips with green nodes. Quantification of the network parameters are shown in Fig. 1D-F. **B)** An image of a P6 mouse head. The white arrow indicates the ventral side of the ear pinna and the blue arrow the tragus that masks the opening to the inner ear. Yellow arrow points at the dorsal side of the ear pinna. **C-E)** (C) A phase contrast image of a dermis exposed ventral side of a P6 mouse ear pinna. (D) The epifluorescence image of anti-LYVE1 stained ventral ear pinna shows ventral superficial LV networks invading from the base (yellow arrow) and the edge (white arrow). In C and D blue arrow points at the tragus similar to B). E) The ear pinna shown in (C-D) was cleared, imaged with confocal microscope, and depth color-coded. Red LVs grow at the superficial ventral dermis. White arrows point at the connection to the deep dorsal LV network (yellow/green). The original stack is shown in Supplementary Movie 2. Images shown in (C-E) are representative of n=5 ear pinna in 5 mice. **F)** Shows a drawing of the lymphatic endothelial sub-trees growing on the superficial ventral dermis in P6 and P8 ear pinna. Each sub-tree is indicated with a unique color. Images are representative of n=5 P6 and n=5 P8 ear pinna and mice. **G-I)** (G) Shows anti-LYVE1 (red) and anti-VEGFR3 (white) stained P21 ventral ear pinna dermis. Yellow arrows in zoom-in images indicate LYVE1- (pre-)collector segments. Examples of full stacks are shown in Supplementary Movie 7 and 8. H) Graph shows mean proportion +/- SD of the LYVE1+ lymphatic capillary length of all (VEGFR3+) the LVs at P8 (n=5 ear pinna, representing 5 mice), P16 (n=11 ear pinna, representing 9 mice), and P21 (n=10 ear pinna, representing 9 mice). Two-sided Welch's t-test was used for measuring statistical significance. p=0.0003 (P8 vs. P16), p=0.0004 (P8 vs. P21), and p=0.93 (P16 vs. P21). I) Fluctuation exponents of the lymphatic capillary (LYVE1+) or total LV network (VEGFR3) calculated from P21 ventral networks stained with anti-LYVE1 and anti-VEGFR3 (n=10 ear pinna, representing 9 mice each), showing that both

stainings display minimal spatial fluctuations. p=0.49, two-sided Welch's t-test. Data are shown as mean values +/- SD. **J)** Examples of density fluctuation spectra for a P13 and a P21 ventral networks (dots) and power-law fit (taken from x=10 to 750, lines). Scale bars in A) 1000μm,D-F) 500μm, and G) 1000μm or 500μm (zoom-in). Source data for Supplementary Fig. 1H-J are provided as a Source Data file.

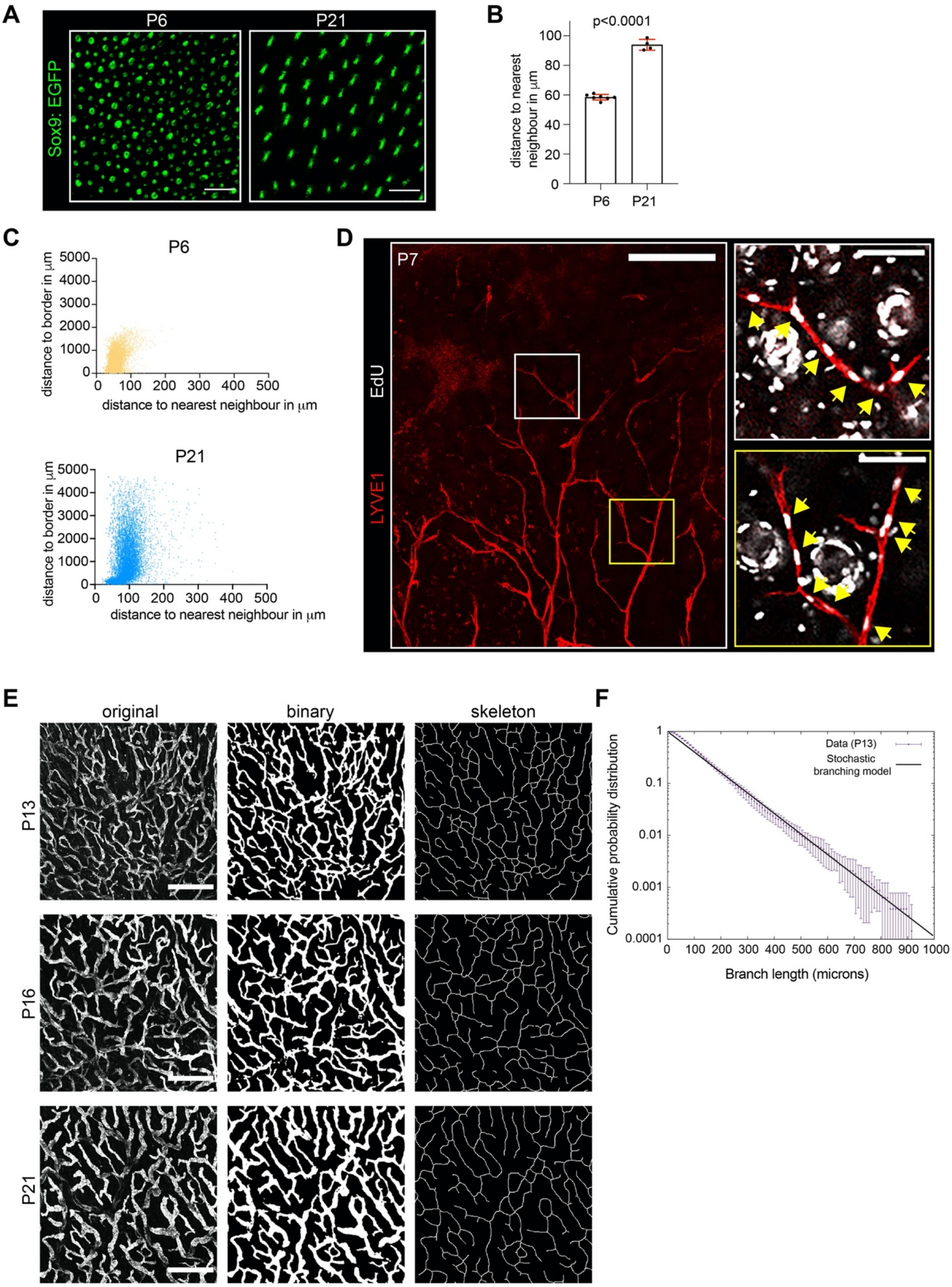

Supplementary Figure 2: Expansion of the ear pinna and the LV network

A) P6 and P21 hair follicles (green) in ventral mouse ear pinna as highlighted by Sox9 promoter-driven EGFP. Scale bars are 200μm. **B-C)** Dot blot shows the mean +/- SD of median distances of hair follicles in each analyzed ear pinna at P6 and P21 (p<0.0001), whereas the scatter blots show the distances of all the hair follicles to their nearest neighbour (x-axes) as a function of distance to the outer border of the ear pinna (y-axes). n=7 ear pinna representing 5 mice (P6) or n=4 ear pinna representing 3 mice (P21). In B) two-sided Welch's t-test was used for measuring statistical significance. **D)** The flattened overview image shows anti-LYVE1 stained (red) superficial LVs invading the ventral dermis at the base of the P7 ear pinna. The zoom-in images show a single optical section of LYVE1+ LVs and EdU (white) that incorporated the DNA upon 4h pulse labeling. The zoom-in image framed in white highlights a tip of an LV tree whereas the zoom-in image, below, framed in yellow highlights a part of the trunk of an LV tree. Yellow arrows point to examples of EdU-positive lymphatic endothelial nuclei. Scale bars for overview and magnified images are 200μm and 50μm, respectively. The shown image is representative of n=5 mouse ear pinna and 5 mice. **E)** Examples of LYVE1 stained (grey) original image, segmented binary image and the corresponding skeleton used for density fluctuation analysis of the LV-networks at P13 (n=5 ear pinna and mice), P16 (n=8 ear pinna, representing 7 mice) and P21 (n=9 ear pinna representing 8 mice). Scale bars are 500μm. **F)** Distribution of branch lengths (defined as the distance from two branch points) in reconstructed P13 lymphatic networks, showing broad exponential-like distributions consistent with the model of stochastic branching (black line). Data are shown as mean values +/- SD (n=4 ear pinna in 4 mice). Source data for Supplementary Fig. 2B-C, and F are provided as a Source Data file.

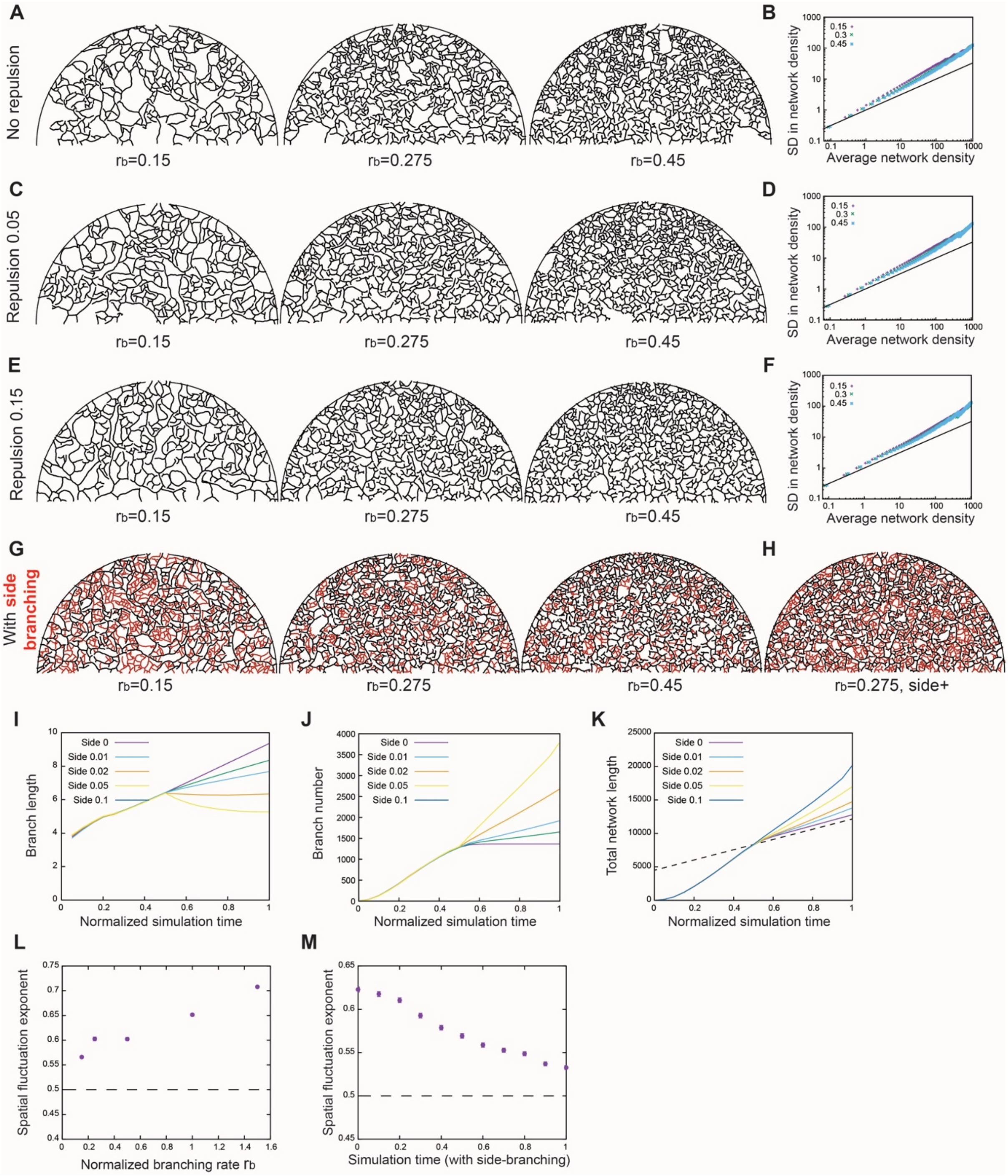

Supplementary Figure 3: Sensitivity analysis for model predictions

A-F) Computational exploration of how different parameters in our model impact the density fluctuation exponent in the absence of side-branching. We explore in particular the effect of different levels of tip-branch repulsion f_s (no repulsion: A-B, intermediate repulsion: C-D, strong repulsion: E-F), as well as the effect of different branching rates (controlling the overall

average density in the network: left to right in each line for increasing branching rate r_b). In all cases, fluctuation exponents are robustly above the minimal value of 0.5 (B, D, F, black line) and consistent with the exponents measured experimentally in wild-type P13 lymphatic networks. **G-H)** Computational exploration of how different parameters impact the density fluctuation exponent in the presence of side-branching. With intermediate values of side-branching (all branches born from side-branching events in red, all others in black), we find that the system becomes rather insensitive to the value of the tip-branching rate r_b, consistent with side-branching being able to rescue low branching rates (G, left to right) – see also Fig. 2E,G. **I-K)** Effect of different values of side-branching rate (different colors) on the final network morphology (giving rise to larger densities), as well as on the temporal evolution of different morphometric parameters. In particular, branch length is expected to linearly increase through time in the absence of side-branching due to overall ear dilation, while the effect can be dampened or even reversed as a function of the side-branching rate giving rise to smaller branches (I). As expected, increasing values of side-branching rate in the second phase of branching also result in increasing branch numbers (J) and total network length (K). **L,M)** Value of fluctuation exponents in simulations under different theoretical assumptions (corresponding to respectively Fig. 2E,G). Fluctuation exponents do not converge towards 0.5 (minimal spatial fluctuations) upon an increase of tip-branching rate (L, calculated from final P21 snapshots), but do rapidly converge towards 0.5 as side-branching proceeds (M, calculated as a function of time/number of side-branching events). Source data for Supplementary Fig. 3L-M are provided as a Source Data file.

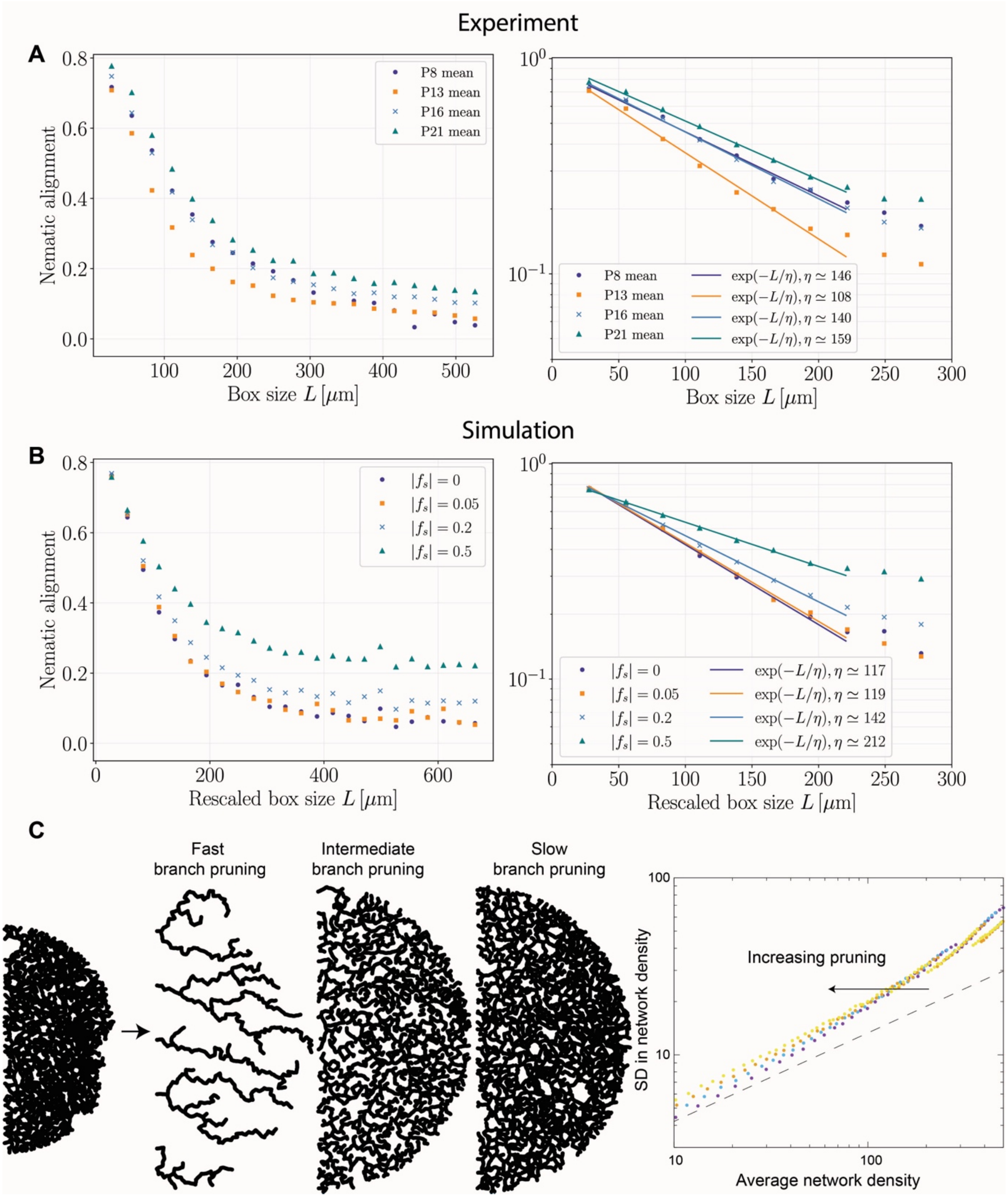

Supplementary Figure 4: Additional analyses of the branching alignment of wild type lymphatic vessel networks and comparisons to the model

A) Nematic alignment given by the scalar order parameter S as a function of box size L averaged over different lymphatic networks at each developmental stage from the experimental dataset (P8, n=4; P13, n=5; P16, n=3; P21, n=2 networks). (Left) At P13 the order parameter decays faster with increasing L compared with that of the other developmental time points. (Right) Up to L=200um the decay can be well-fitted by an exponential with a characteristic length $\eta \simeq 100\mu m$ for P13, and with $\eta \simeq 150\mu m$ for P8, P16 and P21. **B)** Nematic alignment as a function of box size L (in rescaled units) obtained from the BARW simulations with different choices of self-repulsion strength f_s (n=10 networks for each choice). (Left) Networks with small and intermediate self-repulsion, i.e. $|f_s| \leq 0.2$ exhibit a similar decay profile to the experimental data. (Right) For $|f_s| = 0.2$ the characteristic decay length from simulations $\eta \simeq 140\mu m$ is comparable to that of the early and late-stage experimental networks. **C)** Exploration of branch pruning in the simulations. We consider a first phase of side-branching as previously defined, followed by a second phase with a constant probability to prune a given end-branch. Snapshots show representative results for large, intermediate, and small rates of pruning (left). We find that changing the rate of pruning does not impact the spatial fluctuation exponent(right panel). Source data for Supplementary Fig. 4A-C are provided as a Source Data file.

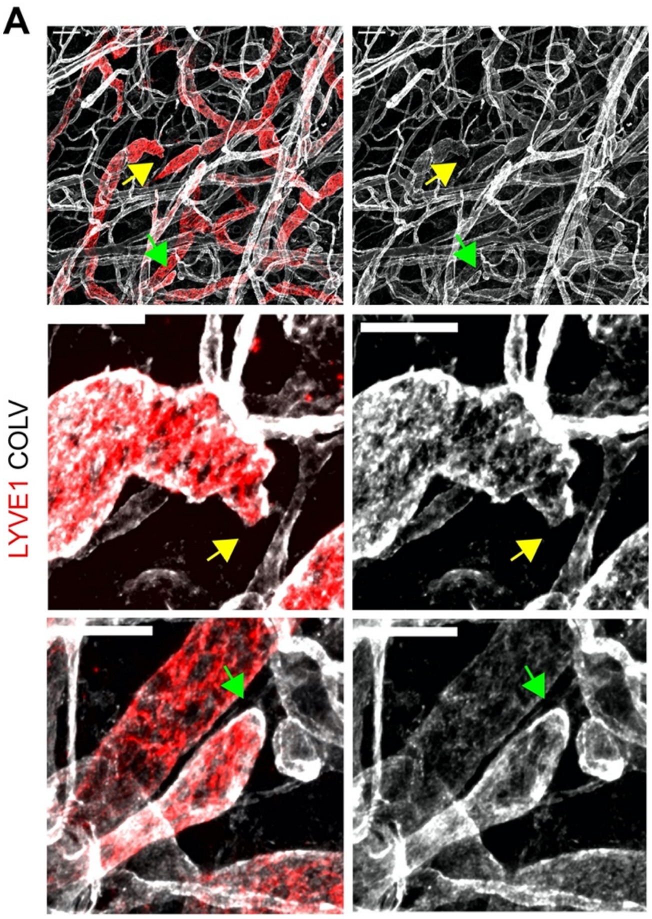

Supplementary Figure 5: Lack of empty basement membrane sleeves at P21

A) LYVE1 (red) and collagen IV (white) stained P21 mouse ear pinna ventral dermis. Arrows indicate two exemplary capillary tips that are also highlighted in magnified images. A representative image of (n=8 ear pinna representing 6 mice) is shown. Scale bars are 100μm in overview and 50μm in magnified images.

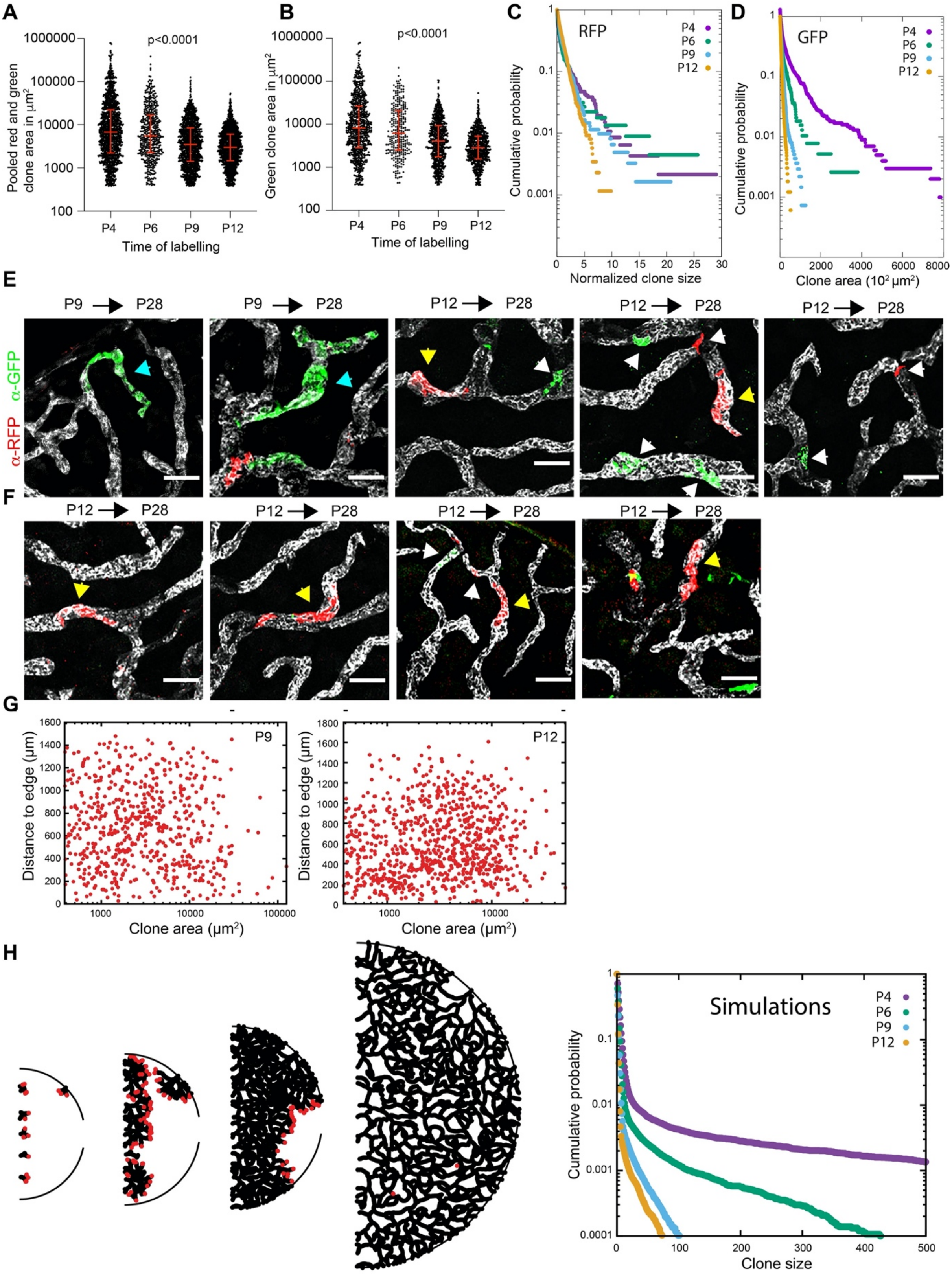

Supplementary Figure 6: Additional analyses of clonal labelling of developing LV networks

A-B) Dot blots show separately the median with interquartile range of A) pooled red and green (P4 n=1423, P6 n=505 , P9 n=1374 , and P12 n=1653 clones) or B) green clone areas (P4 n=835 , P6 n=281 , P9 n=762 , and P12 n=785 clones). Kruskal-Wallis test was used for measuring statistical significance ($p<0.0001$). **C)** Normalized clone size distribution of tdTomato clones (Same data as Fig. 3G.), where the distributions have been rescaled (x-axis) by the average clone size at each time point. This demonstrates that the shape of the distributions is markedly changed between the early and late induction (in particular due to heavy tails in the former, which are not explained by changes in average clone sizes). **D)** Clone size distribution of anti-GFP stained clones, showing similar broad tails at early induction time point as tdTomato clones (see Fig 3G). **E-F)** anti-RFP (red) and anti-GFP (green) stained clones of LYVE1 stained (gray) LV network. The time of 4-hydroxy tamoxifen treatment and collection (P28) is indicated. Cyan arrows indicate uni-clonal branches, white arrows single lymphatic endothelial cells, and yellow arrows lymphatic endothelial clones composed of few cells. Scale bars are 100μm. **G)** Lack of correlation between the clone area (x-axis) and distance of the clone center of mass from the ear edge (y-axis), both for P9 and P12 inductions. This supports uniform growth of the network at later time points. Each dot represents a clone. Data shown in A-G is related to the data set shown in Fig. 3 and consists of: P4, 12 ear pinna and 6 mice; P6, 7 ear pinna and 4 mice; P9, 5 ear pinna and 5 mice; and P12, 10 ear pinna and 5 mice. **H)** Sensitivity of the model prediction to changes in the initial conditions, where we have considered a sixth initial tree growing from the ear tip (left). This produces networks that are highly similar to those shown in Fig. 2, as shown quantitatively by similar simulated clone size distribution (right panel, compare with Fig. 3F, see also Supplementary Movie 12). Source data for Supplementary Fig. 6A-B, D and G are provided as a Source Data file.

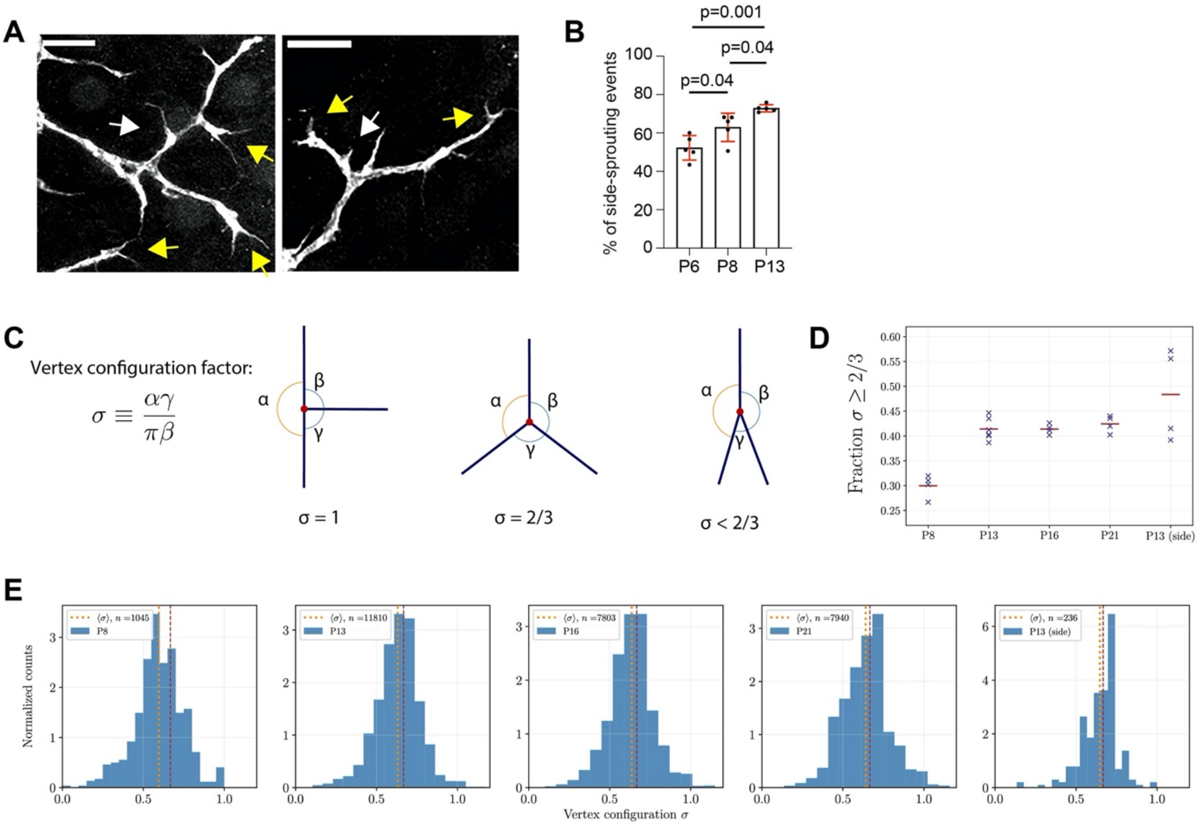

Supplementary Figure 7: Both tip-splitting and side-branching of existing vessels contribute to LV network build-up

A-B) LYVE1 staining of a P6 ventral ear pinna dermis. Yellow arrows indicate tip-splitting events and white arrows sprouting of an existing LV. Scale bars are 50µm. A quantification shows the mean +/- SD percentage of side-branching events (sprouting of an existing vessel) of all branching events (side-branching + tip-splitting) at P6 (n=5 ear pinna and mice), P8 (n=5 ear pinna and mice), and P13 (n=5 ear pinna, representing 3 mice). Two-sided Welch's t-test was used for measuring statistical significance. p=0.04 (P6 vs. P8), p=0.001 (P6 vs. P13), p=0.04 (P8 vs. P13). These samples were part of the data set also shown in Fig. 1B, Supplementary Fig. 1C-F and H and Supplementary Movies 2-6. **C)** Schematic illustration of the vertex configuration factor σ which quantifies the angular configurations around branching events by reweighting the ratio of the largest angle α to π with the ratio of the two smaller angles γ

to β. The drawings illustrate values of σ for different configurations: σ=1 for perfectly lateral branching modes (left), σ=2/3 for symmetric configurations (middle), and σ<2/3 for "fork-like" bifurcations (right). **D)** Fraction of branching configurations with σ>2/3 relative to all calculated σ values from entire LV networks across different developmental stages. A larger fraction indicates an increasing occurrence of symmetric and lateral branching modes as illustrated in panel C. The column "P13 (side)" shows vertex configuration factors σ for manually identified side-branching events (n=236) from P13 datasets. **E)** Cumulative histograms of calculated vertex configuration factors σ across different developmental stages, and for the manually identified P13 side-branches (rightmost panel). Dotted (orange) and dashed (red) vertical lines indicate the mean of samples and the value σ=2/3, respectively. Source data for Supplementary Fig. 7B and D-E are provided as a Source Data file.

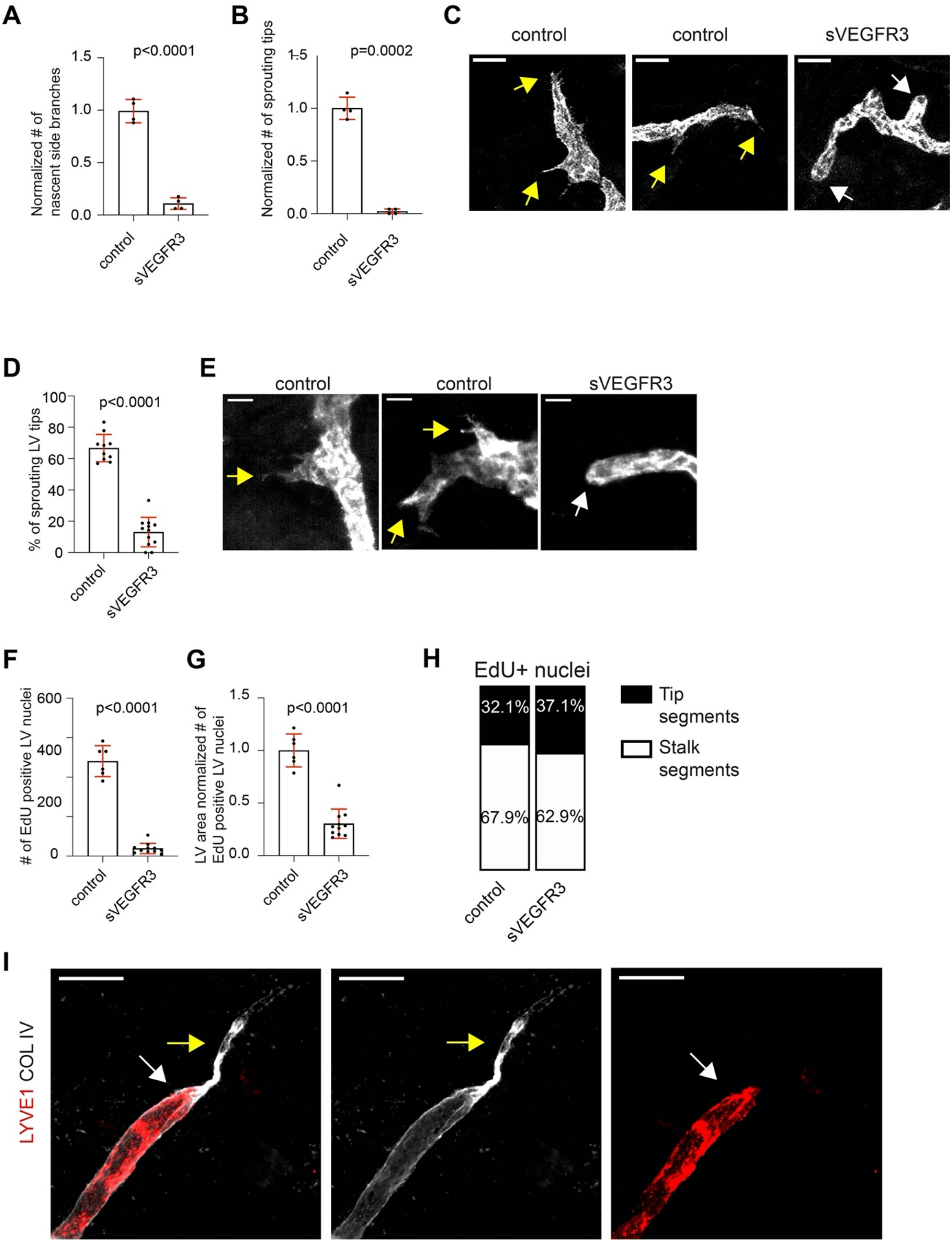

Supplementary Figure 8: Sequestration of VEGF-C attenuates lymphatic endothelial side-branching, tip cell phenotype and proliferation

A-C) In A) a dot blot shows the mean number +/- SD of A) nascent sprouts of existing LYVE1+ lymphatic capillaries (side branches) ($p<0.0001$) and B) percent of sprouting lymphatic capillary tips ($p=0.0002$) in control (n= 4 ear pinna and mice) and ligand trap treated (n= 4 ear pinna and mice) mice, at P13, i.e. only two days after treating mice with VEGF-C/D ligand trap encoding AAV-vector. Samples have been normalized to the size of the analyzed area and then the average of controls has been set to 1. C) LYVE1 staining of P13 ventral ear pinna show invasive tip cell and nascent sprouting of existing LVs (yellow arrows) in control mice, whereas the tips are blunted in VEGF-C ligand trap treated mice (white arrow). **D-E)** In D) a dot plot shows the mean percentage +/- SD of LYVE1+ lymphatic capillary tips that display a pointy tip cell phenotype upon control (n= 10 ear pinna, representing 6 mice) or VEGF-C ligand trap (n=12 ear pinna representing 6 mice) treatment from P11 to P16 ($p<0.0001$), i.e. the samples were collected at the time of ongoing LV growth. E) LYVE1 staining of P16 ventral ear pinna show invasive tip cell/sprout phenotype (yellow arrows) in control mice, whereas the tips are blunted in VEGF-C ligand trap treated mice (white arrow). **F-H)** Dot plot shows mean (+/- SD) F) absolute ($p<0.0001$) or G) LV area ($p<0.0001$) normalized (average of controls in each experiment set as 1) number of EdU+ nuclei in the LYVE1+ lymphatic capillaries upon control (n=6 ear pinna, representing 5 mice) or VEGF-C ligand trap (n=11 ear pinna, representing 6 mice) treatment (from P11 to P16). H) A Stacked bar graph shows the proportion of EdU+ LV nuclei in the tip segments and stalk segments of the control (n=4 ear pinna, representing 4 mice) or VEGF-C/D ligand trap treated (n=7 ear pinna, representing 4 mice) mice. **I)** LYVE1 (red) and collagen IV (white) staining shows an empty basement membrane sleeve (yellow arrow) of an LV in an ear pinna of mice treated with sVEGFR3 from P11 till P21. Images are representative of n=4 ear pinna, representing 3 mice. Scale bars are 20μm. Two-sided Welch's t-test was used for measuring statistical significance in A-B, D, and

F-G. Source data for Supplementary Fig. 8A-B, D, and F-H are provided as a Source Data file.

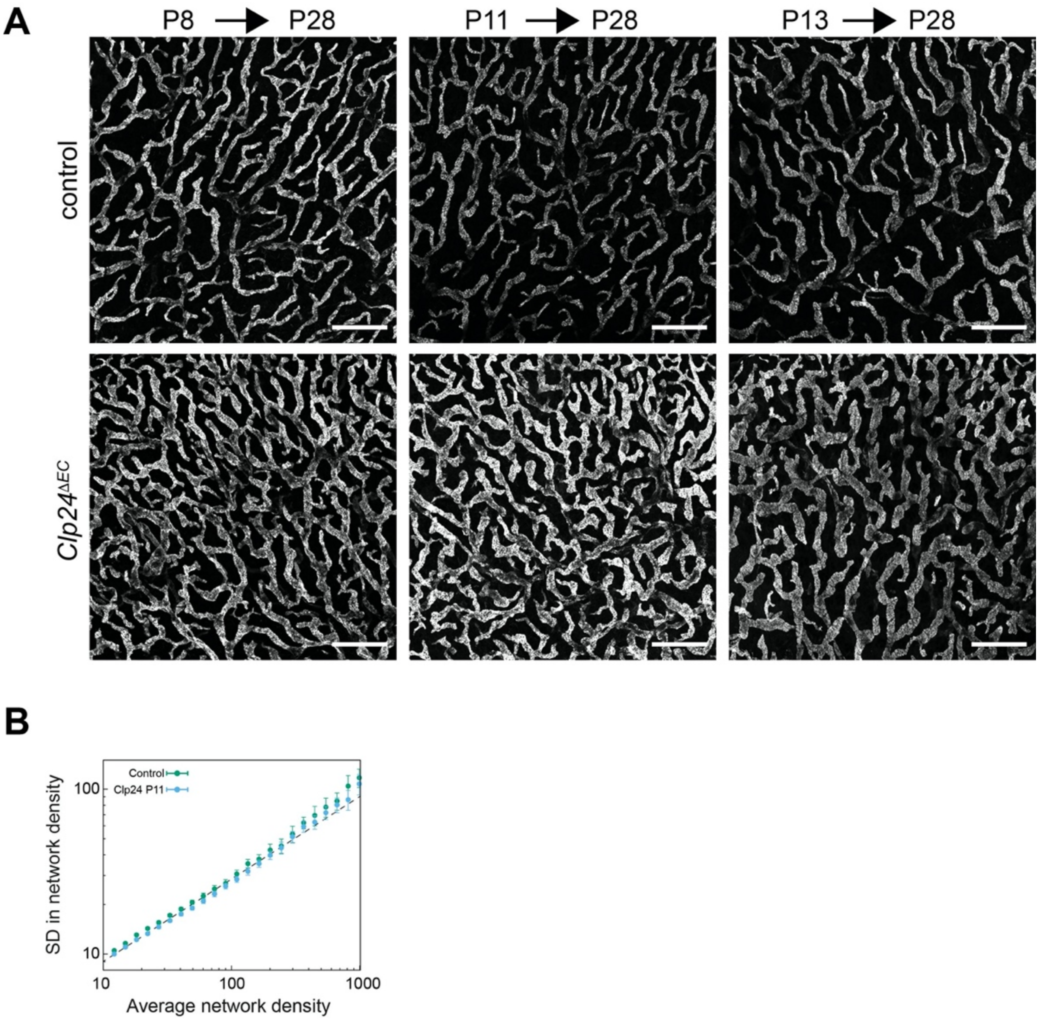

Supplementary Figure 9: *Clp24$^{\Delta EC}$* lymphatic vessel networks show increased side-branching

A) Additional examples of LYVE1-stained control and *Clp24$^{\Delta EC}$* networks analyzed in Fig. 4L and S9B. Mice were treated with 4-hydroxy tamoxifen from P8, 11 or 13 onwards and ears were collected at P28. Scale bars are 500μm. **B)** Quantification of the efficiency of space-filling, measured by the amount of spatial density fluctuations, for control (n=5 ear pinna) and P11- 4-hydroxy tamoxifen-treated *Clp24$^{\Delta EC}$* (n=6 ear pinna) networks for P21 mouse-ear ventral dermis, representing 3 mice each. The dashed line indicates a minimal fluctuation exponent of ½. Source data for Supplementary Fig. 9B are provided as a Source Data file.

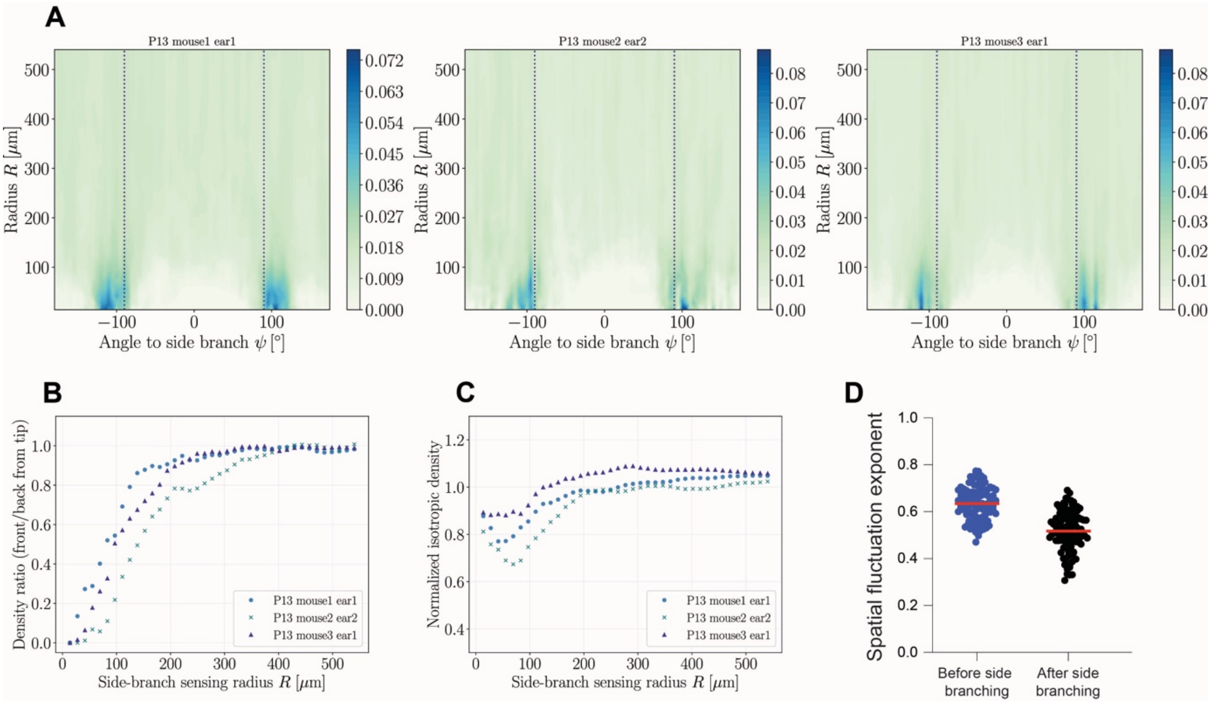

Supplementary Figure 10: Additional analyses of density-dependent side-branching mechanism at P13

A) Angle between the side-branch initiation vector (see illustration in Fig.5C in the main text) and neighboring branches as a function of sensing radius *R* for each ear. The color bar represents the relative frequencies of ψ, dotted vertical lines correspond to ψ = ±90°. **B)** Ratio of probabilities of neighboring LVs in the "front" (with |ψ| < 45°) vs "back" (with |ψ| > 135°) of the side branch initiation vector exhibits a conserved length scale of sensing radius *R*≃200µm for the individual ears. **C)** Ratio of isotropic densities around side branches ρ_s to densities ρ_r around random points on the networks for different values of the sensing radius *R*. **D)** Spatial fluctuation exponent in simulations before and after the phase of side-branching (same simulations as Fig. 5A, under the directional and isotropic sensing). Simulation data exhibit the statistical variance of the fluctuation exponent due to the finite system size. Interestingly, the range of the noise attains similar values as in the experimental data, see Fig. 2B and S1I. Source data for Supplementary Fig. 10A-D are provided as a Source Data file.

Supplementary Theory - Self-organized and directed branching results in optimal coverage in developing dermal lymphatic networks

Contents

1 Models of lymphatic network formation as a branching and annihilating random walk **22**

1.1 Description of the model of branching and annihilating random walks 22

1.2 Incorporation of tip-vessel repulsion in the model 23

1.3 Incorporation of branch pruning in the model 25

1.4 Incorporation of side-branching in the model 25

1.5 Incorporation of ear growth in the model 26

1.6 Simulation of lineage tracing in the branching and annihilating random walk model 26

2 Parameter fitting and model predictions **28**

2.1 Parameter fitting 28

2.2 Model predictions 29

3 Local regulation of side-branching: modelling strategy and associated quantification **30**

3.1 Modelling of side-branching initiation 30

3.2 Data analysis and model comparison 31

3.3 Morphometric analysis of branching points 32

In this Supplemental Theory Note, we provide additional details on the modelling approach, as well as sensitivity analyses on how different models and parameters affect the structure of in silico lymphatic networks compared to our in vivo dataset.

1 Models of lymphatic network formation as a branching and annihilating random walk

Our data shown in Figure 1 supports that post-natal lymphatic network formation in the mouse ear pinna dermis proceeds via two complementary processes: a first phase of invasive branching morphogenesis (from P4 to P10-11), where a small number of tree-like networks invade the ear via iterative rounds of branching and elongation, as well as a second phase (P11-P21), where the ear is already filled by lymphatic networks, but where we still observe an increase in the total number of branches in a given ear (Figure 1).

1.1 Description of the model of branching and annihilating random walks

Given that the first phase of branching morphogenesis gives rise to networks which are highly stochastic from one mouse to the next, as well as inefficient at space-filling (Figure 2), we first consider the simplest self-organized model for this process as a branching and annihilating random walk. This type of models, which has been proposed to understand branched organ morphogenesis (such as mammary gland, kidney or pancreas) as well as neuronal morphology [1], considers that branch structures emerge via a set of simple rules. Actively growing tips are responsible for the bulk of the organ growth, and drive local ductal elongation by undergoing a persistent random walk (note that we do not model the details of the mechanics involved in this, which presumably requires both cell migration and proliferation). Furthermore, a given tip has at any time point a given probability to branch into two tips (branching random walk). Finally, these two rules alone would give rise to an uncontrolled exponential growth of the structure, so that we need to posit a mechanism for tip termination/annihilation. Interestingly, ear lymphatic networks form largely in 2D, with very little overlap between vessels, and in particular show consistently tips that are arrested in growth at a given, small distance, from another vessel. This is close to previous observation in organs such as mammary gland, and can be modelled by density-dependent tip termination, where a given active tip irreversibly becomes inactive (i.e. cannot elongate or branch anymore) when it gets too close to a duct. These three rules have been shown to be enough to give rise to self-organized branched organ growth at a set density [2].

More precisely, considering active tips as particles A and inactive vessels left behind by tip elongation or terminal as particles I, the model has the following parameters (note that for simplicity, we consider all tips to have the same underlying parameters):

- tips elongate at an instantaneous speed v_0, with angular diffusion D_r (persistent random walk), giving rise to vessels in their wake. ($A \to A + I$ process)

- tips branch into two tips probabilistically at rate r_b ($A \to A + A$ process). Note that this means that the branch length will be exponentially distributed, with characteristic length scale related to v_0/r_b.

- tips terminate irreversibly if they are within a radius R_a of other vessels. ($A \to 0$ process)

We perform stochastic numerical simulations of this process, where at every time interval dt, a given tip i moves by a distance $v_0 dt$ in its preferred direction θ_i. This angle θ_i changes gradually at every time step (persistent random walk), by the addition of a small random component picked from a uniform distribution between $[-\delta\theta, \delta\theta]$, with $\delta\theta = \pi/10$. At every time point, we pick a random number and compare it to $r_b dt$ to determine whether this tip also branches into two tips. If so, we terminate the tip i and create two new tips with angular direction $\theta_i + \theta_1$ and $\theta_i - \theta_2$, where θ_1, θ_2 are random branching angles picked from a uniform distribution $[\pi/6, 2\pi/3]$. Finally, we check at every time point whether the tip is below a distance R_a of any other vessels, or outside the boundary of the simulations, in which cases we terminate its growth. Note that in two-dimensions, the exact value of R_a has a small influence on the network growth (as long as it is small compared to other length scales) as the probability of two branches to intersect is 1. The exact value of the rotational diffusivity and branch angle distribution also has a mild effect on the overall dynamics [2].

An important feature of this model is that termination/annihilation is irreversible: once a given branched region has no active tips anymore, it is "frozen". This manifests into very large (non-equilibrium) spatial fluctuations, as quantified in analogy to active matter systems from statistical physics, by quantifying the spatial standard deviation vs spatial averages of network density at multiple length scales L. More specifically, we divide a given system into boxes of size LxL, and count for each box the network density ρ_L. We then calculate population-level standard deviation and averages across all boxes ($\sigma(\rho)_L$ and $<\rho>_L$), and plot these two quantifies as a function of one another across many values of L. Equilibrium physics imposes that $\sigma(\rho)_L \propto (<\rho>_L)^\alpha$, with $\alpha = 0.5$, whereas exponents $\alpha > 0.5$ indicate giant fluctuations (note that α must be small than 1, as this corresponds to maximal standard deviation, i.e. all the network to be in one box).

Interestingly, calculating the fluctuation exponent α for the P13 data revealed significant departure from minimal fluctuations (Figure 2A,B), with values of $\alpha = 0.6$ which were fully consistent with previous exponents found for our model of branching and annihilating random walk, across different parameter regimes (Figure 2D-E, Supplementary Figure 3A-F). This qualitatively suggests that this model might be able to capture some features of our data.

1.2 Incorporation of tip-vessel repulsion in the model

In addition to the simple rules for branching and annihilating random walk discussed above, a possible extension of the model, that has been studied in particular in the context of branched neurons, is that tips do not simply terminate in the vicinity of surrounding branches, but also actively avoid them, by processes such as adhesion-mediated repulsion [3] or local gradient sensing [4]. In our framework, this can be captured

by considering that at every time point, a given tip locally senses vessels density in a given radius R_{rep} and adapts its direction of growth accordingly.

More specifically, for a tip i with direction of growth θ_i and position $\vec{r}_i$, we compute the local density vector $\vec{R} = \sum_j \frac{(\vec{r}_i - \vec{r}_j)}{|\vec{r}_i - \vec{r}_j)|} e^{-\frac{|\vec{r}_i - \vec{r}_j|}{R_{rep}}}$, where index j lists all the neighbors of i (for computational convenience, we only calculate it for $|\vec{r}_i - \vec{r}_j| < 2R_{rep}$ given that vessels further away have vanishing contributions). This local density vector $\vec{R}$ is defined such that it points towards regions of low network density (with density being "measured" locally as vessels further contributing less compared to vessels nearby the tip). Then, in the elongation step, instead of updating as before tip coordinates (x_i, y_i) as $(x_i + v_0 dt cos(\theta_i), y_i + v_0 dt sin(\theta_i))$, we instead update it $(x_i + v_0 dt cos(\phi_i), y_i + v_0 dt sin(\phi_i))$, where the angle ϕ_i is calculated by updating θ_i with a repulsive displacement $f_s \vec{R}/|\vec{R}|$ (see [1], for further details and geometrical representation).

Intuitively, this introduces the parameter f_s which quantifies the strength of tip-vessel repulsion and represents the tendency of tips to actively move away from regions of high network density (in a typical radius R_{rep}). However, such repulsive interactions do not change the core dynamics of the first phase of branching morphogenesis. Indeed, the global dynamics of invasive branching morphogenesis by outward propagation of active tips is identical, as is the dynamics of annihilation of tips in the back of the invasive growth front (in 2D, even with repulsion, the geometrical constraints are such that annihilation is always dominant at a given density). The main quantitative differences with repulsion are i) an overall larger network density, as expected from the fact that tips can be partially and locally guided in low density regions prior to termination (see [1] for more details), and ii) an increased local branch alignment driven by repulsion. Here, we reasoned that this latter effect can be used to constrain the amount of repulsion during lymphatic branching morphogenesis.

Indeed, we computed the nematic order as a proxy for local alignment between individual branch segments in both simulations and experimental data. We quantified the nematic order in network regions of size $L \times L$ and its decay as a function of different box sizes L. To prevent confounding effects arising from large density fluctuations of the networks, we constrained the analysis for boxes with branch densities comparable to the global density of the network. In each box, we define a local director vector $\mathbf{n} \equiv N^{-1} \sum_j \mathbf{u}_j$, where $\mathbf{u}_j$ are individual unit vectors oriented along N discrete branch segments in the box (based on coarse-grained skeletonized coordinates). We then determine the scalar order parameter $S \equiv \langle 2\cos^2(\varphi_j) - 1 \rangle$ averaged over all boxes of size $L \times L$, where φ_j is the nematic angle between the unit vectors $\mathbf{u}_j$ and the local director vector $\mathbf{n}$ [5]. For P13, we found that the nematic order decayed with a characteristic length estimated by $\eta \simeq 100\mu$m from an exponential fit $S \propto e^{-L/\eta}$ for small L. Early and late-stage networks P8, P16 and P21 similarly displayed exponential-like decays, with slightly longer decay lengths of $\eta \simeq 150\mu$m, see Supplementary Figure 4. We reasoned that the faster decay of local nematic alignment at P13 compared with those of P8, P16 and P21 might be indicative of extensive side branching taking place at the onset of the second phase of network growth. We then determined the nematic order in simulations with different amounts of self-repulsion as quantified by the repulsion strength f_s. Interestingly, the decay profile from

simulations both with small and intermediate values of self-repulsion ($|f_s| \leq 0.2$) agreed well with those from the P8-, P16- and P21 datasets at comparable rescaled lengths, see Supplementary Figure 4. For a self-repulsion of $|f_s| = 0.2$ the simulations exhibited a decay length of $\eta \simeq 140\,\mu$m, close to the value from the experimental early- and late stage networks.

1.3 Incorporation of branch pruning in the model

To consider the effect of branch pruning (i.e. deletion) in our simulations, we considered that any branch has a finite probability r_{pr} at any given time point to be deleted from the simulations. More specifically, to model the effect of ligand-trap treatment from a specific time point (P11), we ran the simulations with the same parameters as above (Supplementary Figure 4C) and allowed for pruning at different rates r_{pr} after the second half of the simulations. Only a terminal branch can be pruned, otherwise this would lead to disconnected branch segments. Therefore, for large pruning rates, terminal branch points of lower and lower generation numbers can be selected for pruning, leading to very sparse networks with each subtree only consisting of a a few long branches. Importantly, when calculating the network fluctuation at the end of simulations for different r_{pr}, we found only marginal effects on the exponent of fluctuations, as expected given that we randomly delete branches from a network already showing giant fluctuations pre-pruning.

1.4 Incorporation of side-branching in the model

Given the findings described in the main text, we next sought to incorporate the possibility of side-branching into our model. From a mathematical perspective, this drastically modifies the model, as the state of zero tips ($A = 0$) is no longer an absorbing state for the dynamics. Intuitively, this means that the local network density is not frozen to a value dictated by the phase of invasive branching morphogenesis, but can continually evolve and increase throughout time. This type of active side-branching is then expected to influence the global topological properties of the growing tree, as explored mathematically in the past [6], and bears similarities with a recent model of branching-delayed random walks that describes salivary gland development [7].

We first consider the simplest case of random side-branching, i.e. where any inactive particle has a random probability r_s to become active again ($I \to A$) at any time point. When this occurs, we randomly pick a new growth direction θ_i perpendicular to the local vessel that the new tip is emanating from. As the process of tip termination still operates, if we have picked a particle that is in a radius R_a of other vessels, re-activation of growth/side-branching does not occur. Although adding this new process does not qualitatively change the first phase of tip-driven invasive branching morphogenesis (Figure 2D-G and Supplementary Figure 3G-K), it drastically modifies the second phase by driving a continuous increase of branch number even after the entire ear has been filled up (Supplementary Figure 3I,K).

In particular, if we run this second phase over long times, the branching network converges towards a new maximal density which is dictated by the radius of annihilation R_a: all regions of the ear become filled

up until every particle is closer than R_a to a neighbor (so that no side-branching can occur).

1.5 Incorporation of ear growth in the model

So far, we have considered the process of branching morphogenesis in a domain of fixed size. However, we have found evidence that the ear grows homogeneously throughout neonatal development (Figure 1A-B), with roughly linear dynamics (represented by a growth in ear radius $L(t)$ - modelling the ear as a half disk). We incorporate this in our simulations by homogeneously dilating the network at each time point following the global growth dynamics $L'(t)$.

Although this does not significantly change the dynamics of the first phase of branching morphogenesis (given that the speed of network growth is much larger than the speed of ear growth, see Figure 1), it has an important impact on the second phase of side-branching.

Denoting $\rho(t)$ the global density of the network (for instance obtained by dividing the total length of the network by the total ear area $S(t) = \pi L(t)^2/2$), a simple toy model for the time evolution of $\rho(t)$ is:

$$\rho(t)S'(t) + S(t)\rho'(t) = r_s\rho(t)S(t)\,, \tag{1}$$

which indicates that without side-branching the network density simply becomes diluted by an area increase $S'(t)$ (left-side terms), while side-branching at rate r_s can allow the density to increase. Rearranging this equation as $\rho'(t) = \rho(t)\,(-S'(t)/S(t) + r_s)$ shows that there are two limits: for side-branching rate r_s below a critical threshold compared to area growth $S'(t)/S(t)$, overall density is expected to decrease during the second phase, while for large-enough side-branching, overall density can increase despite area growth.

Note that this simplified equation at the coarse-grained level does not take into account the annihilation radius of tips, which prevents density from increasing exponentially even with large side-branching rates. At a more discrete level, if ear growth occurs while the network is already maximally packed, this will gradually dilate some regions where some vessels will become further away to their neighbors than the annihilation radius R_a allowing for local compensatory growth and an active maintenance by side-branching of a highly packed structure.

1.6 Simulation of lineage tracing in the branching and annihilating random walk model

To simulate our lineage tracing experiment, we re-ran our simulations while irreversibly labelling all particles with unique ID at a given time point (systematically time-matched to the experiments as discussed above). When a tip subsequently grows at speed v_0 (either terminal tips from the first phase or those arising via side-branching), all the inactive vessel particles it gives rise to inherit the same ID, see Fig 3C for typical simulations matched for the P6 and P12 experimental inductions, with a small fraction of clones labelled in red.

In the limit case of no side-branching, such a model gives rise to highly bimodal distributions in clone sizes: all inactive particles labelled give rise to clone sizes of 1, while labelled tips give rise to large clones

spanning entire regions of the network (with however highly stochastic and broadly distributed sizes, as expected from a process of neutral competition between different tips during a branching and annihilating random walk). Furthermore, lineage-tracing during the second phase (after the network has reached the edge of the ear) is predicted not to give rise to any large clones, given that all tips are extinct at this point.

In the limit of non-zero side-branching ($r_s > 0$), the picture is slightly different, as clones can grow even in the second phase when inactive/vessel particles are selected for a side-branching event. However, the picture of wide, bimodal distributions still holds: early lineage tracing is predicted to give rise to a population of very large clones spanning large branching regions (tips being labelled in the first phase of branching) and a population of small clones (either one cell or few cells consisting of local side-branching events). Late lineage tracing on the other hand is predicted to give rise only to this second population of small local clones.

Importantly, this prediction is close to what we observed in our four time points of lineage tracing. Note that we make the typical assumption from the literature that confetti labelled has a time delay of 1.5 day [8, 9], and include this when matching the time points between model and data. When lineage tracing is performed during the first phase of branching morphogenesis (P4 and P6), we observe a small subpopulation of broadly distributed clones spanning entire cohesive regions of the network. However, these clones are absent in the later time points (P9 and P12), although we do observe small branch segments being labelled, supporting our side-branching hypothesis (Figure 3A,D).

Although this captures qualitatively the clonal distribution, one limitation with this model is that it gives rise to most clones being of 1 cell size (i.e. cells that were not tip-cells during the simulation). This is however not realistic as proliferation does occur in lymphatic networks (Supplementary Figure 8), as evidenced by the fact that vessel width increases in time (Figure 1A). This increase in vessel width is the reverse of ear growth without lymphatic vessel proliferation, which would be expected to cause vessel thinning. Thus, for the distributions displayed in Figure 3F, we assume a small constant base line proliferation rate of all cells of r_d (we choose $r_d T = 2.5$ so that every cell divides a few times during the simulation, in analogy with a division time of a few days). We note that such implementation of division (i.e. locally duplicating particles) does not change the rest of the dynamics, and is computationally costly so we only include it in this part of the analysis. With such division rate, we find that clonal distributions are characterized, at small clone size, by an exponential distribution (as expected from cells with stochastic division patterns [10], as observed in the data, see Figure 3G).

We note that further refinement of the model could be possible. In particular, we assume here that there is a single cell per tip, so that labelling this one cell results in large monoclonal vessels. Although this is broadly consistent with the data, showing indeed that large network regions are monoclonal in P4/P6 inductions, one could consider that "functional tips" are a unit consisting instead of several cells which can move and dynamically exchange position (consistent with experimental observations [11]). As shown previously, this would still give rise to monoclonal network regions after a period of clonal competition, with the length of this period (seen in networks as regions with partially labelled branches prior to full monoclonal

conversion) being dependent on the number of functional tip cells [12].

2 Parameter fitting and model predictions

In this section, we detail how key parameters were fitted in the model based on data, as well as the qualitative and quantitative predictions that the model can subsequently make.

2.1 Parameter fitting

We first estimate the dynamics of ear growth, modelled as a half-disk of radius $R(t)$, which grows in time from $t = 0$ to $t = T$ at the end of simulation (T can also be arbitrarily used to set the time scale of the system). The initial radius at P4 can be used to set a length scale in the system, so we set it in the simulations to $R(0) = 100$, and parametrize it according to linear growth (see Figure 1) as $R(t) = R_0(1+\alpha t/T)$, where α is the relative expansion at the final time point (P21) compared to the initial time points (P4-P6), which we estimate to $\alpha = 1.7$. The speed of tip elongation v_0 has a negligible influence in the simulations, as it rescales the dynamics of the system but not its global evolution, so we take $v_0 = 1$.

On the other hand, the branching probability r_b is a key metric as it directly impacts on branch length as well as network density. Experimentally, we observe that average branch length l at P13, when rescaled by ear radius R at that time point, is equal to $l/R \approx 0.037$, which we use in the simulation to fit $r_b = 0.275$ as it gives the right value of l/R at the mid-point of the simulation. As mentioned above, the radius of annihilation has a weak impact on the dynamics, but we estimate it based on the typical distance between terminated tip and the nearest vessel (typically a fraction of the branch length l in our data), leading us to take $R_a = 3$ in our simulation units. For the repulsion radius R_{rep}, we tested different values for it and found that values much smaller than the typical branch length l result in no changes in the overall network morphology (as repulsion is too local to take place prior to annihilation), while values much larger than branch length were unrealistic given known mechanisms for repulsion (such as local mechanical sensing or local gradient formation typically restricted to a few hundred microns), so we took $R_{rep} = 10$, i.e. on the order of a branch length. Constraining the value of the repulsion strength f_s however is more straightforward, as we found (as described in the section above) that we could use the local nematic order to estimate it, with very large values of f_s overestimating the range of branch alignment, while absence of repulsion ($f_s \approx 0$) underestimated it. We thus took $f_s = 0.2$ based on this analysis.

As initial condition, we positioned five tips regularly spaced at the edge of the ear (which itself is delimitated by the curves $y = 0, y = R sin\theta$ with $\theta = [0,\pi]$), so that the tip coordinates are ($y = 0$ and $x = -2R_0/3, -R_0/3, 0, R_0/3, 2R_0/3$. Each tip points perpendicular to the edge $\theta = \pi/2$, mirroring the experimental conditions of P4 of a few initial trees driving branching morphogenesis (we note that the resulting structure is largely insensitive to this initial condition given the self-organization potential and stochasticity associated with each tip growth).

To directly compare between different time points in the model and experiment, we simply took P4 as our time point $t = 0$ and P21 as our time point $t = T$, and linearly extrapolated in between the two (for instance the middle time point P12.5 corresponding to $t = T/2$).

2.2 Model predictions

With this set of parameters, the model could reproduce a number of important features of the data. Firstly, at the qualitative level, the core assumptions of the model (stochastic branching, tip termination at a small distance from neighbouring vessels) matched well with the non-stereotypical structure of the lymphatic networks in mice, characterised by constant density and front-like propagation of tips during the first phase of branching (as evidenced in P4-P8 in Figure 1). Furthermore, beyond the fact that the model produced networks of constant overall densities after the first phase, the model was characterized by extensive spatial fluctuations (large exponents in the range of $0.6 - 0.7$) in close agreement with the P13 data (Figure 2A,B). At a more quantitative level, we found that i) the local nematic correlation function of vessels was well-described by our simple model of small levels of tip-vessel repulsion during branching morphogenesis (Supplementary Figure 4A,B), ii) the average number of branches per ear after the first phase of branching (P13) was close to the experimentally observed one (Figure 1D, Supplementary Figure 3J) and iii) as described above, clone size distribution showed similar functional shape and time-dependency in model and data (Figure 3F,G).

Perturbation experiments were also found to be in agreement with our theoretical framework. In particular, consistent with the fact that side-branching is the core mechanism responsible for the decrease of giant density fluctuations in the second phase of branching, we found that side-branching inhibition (which could be achieved either by Vegf-c mutant or targeted inhibition of sVEGFR3 from P11 onwards - see Figure 4A-J) results in large fluctuation exponents ($\alpha = 0.6 - 0.7$) as predicted in the model with side-branching (first-phase). More specifically, we modelled slightly differently each condition to reflect the timeline of experimental treatment. For Vegf-c mutant, the entire time course of branching morphogenesis is expected to be affected. Therefore, we ran simulations with no side-branching and a branching rate of 25% compared to wild-type (based on Figure 4G), predicting much larger fluctuations as in experiments (Figure 4H-J). For the ligand-trap, targeted inhibition of sVEGR3 was performed at P11, so we keep all parameters as in WT until P11, and then continue to run simulations with no branching (side or tip-branching, leading to regions at the ear edge not yet filled, as seen in data, compare Figure 4D and 4A). Although as shown in Figure 4C, pruning does not significantly affect density fluctuations, we still included it in the model after P11 (to fit the experimental reduction to 40% seen in Figure 4B), as the number of branches already made at P11 in the model would otherwise exceed its experimental value at P21. This also predicted enhanced fluctuations as in the data (Figure 4C, E).

Finally, from a theoretical perspective, we also would predict that increasing branching rate (r_b) via Cpl24 mutants should not qualitatively change the resulting networks, beyond an overall increase in branch

density. Indeed, Cpl24 mutant networks were still characterized by local tip termination in the vicinity of vessels, as well as minimal fluctuation exponents ($\alpha \approx 0.5$, consistent with the theoretical argument that this is the minimal value for spatial fluctuation exponent).

3 Local regulation of side-branching: modelling strategy and associated quantification

Finally, in this section we provide more information on Figure 5, in particular the different types of models we considered for the local regulation of side-branching, as well as quantifications done in the data to test these different models.

3.1 Modelling of side-branching initiation

The simplest implementation of side-branching is by taking an equal probability r_s for any vessel/inactive particle to become an active tip (if it doesn't sit within distance R_a of another vessel). Although such side-branching event can yield to a decrease in giant number fluctuations (as seen in Figure 5A,B), we found quantitatively that this would require a very large increase in the number of branches in the second phase of branching, with typically a two/three-fold increase in branch number required (Figure 5B).

This was in stark contrast to our data, which showed that the reduction in giant density fluctuation exponent α was concomitant with only a $20-30\%$ increase in branch number from P13 to P16/21. We note that this $20-30\%$ increase could be slightly under-estimated due to the possibility that side-branching could have already started between the time point at which the lymphatic network reaches the edge of the ear (P10/11) and the measurement time point of P13. However, this number is still constrained by the fact that networks at P8 already consist of around 700-800 branches despite still low coverage (meaning that the branch number around P10-P11 should be close to P13).

Given previous reports that branching could be induced and/or guided by local factors such as the concentration profile of diffusible factors or of local hypoxia [13, 4, 2], we then explored the idea that optimization of space-filling properties could be achieved in a much more parsimonious manner by rendering side-branching events dependent on the local environment of a branch. We thus explored two, non-mutually exclusive, classes of hypotheses on how this could be achieved in vivo:

- side-branching rate r_s being dependent on the **local network density**. In this hypothesis, a vessel cell/particle i locally measures the average density ρ_i around it (defined as the number of particles in a given radius R_{rep} divided by the area πR_{rep}^2), and has a rate of side-branching inversely related to local density. Many types of numerical implementations are possible for this, including smooth variations of side-branching rate r_s as a function of ρ_i, although for the sake of simplicity, we simply take it as binary: below a critical local density ρ_c, side-branches emerge at rate r_s while these are prevented ($r_s = 0$) above the critical density threshold. In the numerical implementation of simulations, we

thus, as before, iterate over all inactive particles, calculate local density and pick a random number to be compared to $r_s(\rho_i)$, which determines whether branching occurs or not. As in the case of purely constant side-branching, we then pick an orientation θ_i for the new tip as either perpendicular directions compared to its vessel.

- side-branching rate r_s being dependent on the **local network density gradient**. In this hypothesis, a vessel cell/particle i is not sensitive to absolute local densities, but can read local density gradients. Density gradients are defined in the exact same way as the implementation of repulsion that we describe above: we calculate the vector $\vec{R} = \sum_j \frac{(\vec{r}_i - \vec{r}_j)}{|\vec{r}_i - \vec{r}_j)|} e^{-\frac{|\vec{r}_i - \vec{r}_j|}{R_{rep}}}$ which is a weigthed average of all the vectors linking a particle i to its neigbors, and points towards regions of low densities. Vectors with large norms $|\vec{R}|$ indicate regions where density gradients are large, and we therefore as above make side-branching probability $r_s(|\vec{R}|)$ non-zero only when the repulsion vector is above a threshold $|\vec{R}|_c$. In this implementation, the orientation θ_i of the new tip is taken parallel to $\vec{R}$.

Note that in both cases, we assume that the length scale at which cells measure local density (or local density gradients) is R_{rep}, an assumption that could of course be lifted, but is the simplest given the similarity between density-sensing in tip repulsion and density-sensing in side-branching events. As in the case of tip repulsion, taking R_{rep} too large (compared to branch length) would not convey any useful information for side-branching as density would simply converge to its average value, while taking R_{rep} too small would also not give useful information on a cell branching neighborhood.

Interestingly, we found that both implementations of the model drastically increased the speed at which networks converged to minimal fluctuations (characterized by exponents of $\alpha \approx 0.5$). In particular, under both assumptions, low exponents close to 0.5 could be achieved with relatively mild increases in branch number (typically $50 - 100\%$. Furthermore, combining both models of density sensing (i.e. for instance by having the magnitude of r_s dependent on local density ρ_i, but tip growth direction being picked in the direction of local density gradient $\vec{R}$) yielded even better results, with optimal coverage being achieved simply by increasing branch number of 200-300, a number close to the one found in our experimental data (see Figure 1D, 2A,B and 5A,B).

3.2 Data analysis and model comparison

To verify these modelling assumptions on side-branching probability being dependent on local density and/or local density gradients, we examined more closely the P13 network dataset, as this is the time point at which we assume from the model that optimization by side-branching would be most important. As discussed in the main text, we could manually locate a number of side-branching events by identifying nascent arrow-head-like branches (Figure 5C). We confined our analysis on the shortest sprouts to prevent potential confounding effects. We performed whole-network reconstructions by skeletonizing the binary images using scikit-image library [14] and the Skan module [15] to extract vector representations of branch segments [1]. We then

extracted the coordinates of the manually labelled side-branches and used the corresponding skeletonized vectors to proceed with the analysis on their location and direction.

First, we examined the average local network density $\rho_s(R)$ around side branches in a neighborhood of radius R to address the question whether the side-branching events occurred more frequently in sparse network regions. As a control, we also calculated average network densities $\rho_r(R)$ around randomly selected points on the network. Comparing these metrics we found that up to a length scale of $R \simeq 200\,\mu$m side-branch locations indeed corresponded to a lower density of branches (see Figure 5D).

We then asked whether local density *gradients* also had an influence on the side branch statistics. To examine this, we first defined the angle ψ between the side branch orientation vector and vectors connecting the root of the side branch to its neighboring branch segments (extracted from the skeletonized coordinates), as illustrated in Figure 5C. By classifying the neighbors within a radius R as a function of ψ, we could then explore the relative fraction of neighbors with a given angle ψ to the side branches (averaged from $n = 135$ manually labelled side-branch locations) (Figure 5E). Finally, this allowed us to define the conditional frequencies of neighbors $P(|\psi| < \frac{\pi}{4})$ and $P(|\psi| > \frac{3\pi}{4})$ with an angle ψ smaller than $\frac{\pi}{4}$ and larger than $\frac{3\pi}{4}$, respectively, which we used to identify neighbors located opposite to (i.e. in the "back") and in the same direction (i.e. in the "front") of the side-branch orientation vector. Looking at the ratio of these two conditional frequencies $P(|\psi| < \frac{\pi}{4})/P(|\psi| > \frac{3\pi}{4})$ we then found that it exhibited a clear transition from 0 to 1 at length scales of $R \simeq 200\,\mu$m. Qualitatively, this means that below this length scale, nascent side-branches are much more frequently found pointing towards regions of low density than regions of high density. As a control, we performed the same calculation on the dataset of randomly chosen coordinates (n=500), where we defined angles with respect to a vector perpendicular to the chosen vessel segment, and found that this metric was conserved as 1 at all length scales (Figure 5F), confirming that the trend found above is specific to density-sensing. Interestingly, we note that both sensing mechanisms (based on absolute density and density gradients) seem to be operating up to similar length scales of a few hundred microns (similar to typical length scales for the decay of a number of diffusible signals, [2]). How such cues are generated and integrated across scales to give rise to branching events remains to be investigated in the future [16, 4].

3.3 Morphometric analysis of branching points

To characterize the morphologies of tip- vs. side-branching events over different developmental stages, we defined a scalar parameter that quantifies the angular configuration of a (3-valent) vertex. We designated the angles at a branching junction as α, β and γ, with α being the largest angle and γ being the smallest. The vertex configuration factor is then given by the ratio $\sigma \equiv \frac{\alpha\gamma}{\pi\beta}$. In the case of purely lateral side-branching with $\alpha = \pi$ and $\gamma = \beta$, this leads to $\sigma = 1$, and for perfectly symmetric branching with $\alpha = \beta = \gamma$ we have $\sigma = 2/3$ (see illustrations in Supplementary Figure 7C). Fork-like bifurcations with a well-defined small angle $\gamma < \beta$ generally result in $\sigma < 2/3$. It is important to note that when $\alpha = \pi$, any value between 0 and 1 can arise, but $\sigma \geq 2/3$ is only possible when the angle γ is close to β. Therefore, we decided to use $\sigma \geq 2/3$ as

a threshold to identify non-fork-like bifurcation events.

We then located all branching junctions in the skeletonized coordinates of the entire branched networks at P8, P13, P16, and P21, and quantified their vertex configuration factors using a custom-made script. We determined the angles by examining a small region of $\sim 8\mu$m surrounding the branching vertex. In addition, we analyzed the vertex configuration factors of manually identified side-branching sprouts at P13. The fraction of junctions with $\sigma \geq 2/3$ was highest for the P13 side-branching dataset and lowest for the P8 whole-network data, indicating that symmetric or lateral side-branching configurations are more likely to occur than fork-like tip bifurcation events at later developmental stages (see Supplementary Figure 7D-E).

References

[1] Mehmet Can Uçar, Dmitrii Kamenev, Kazunori Sunadome, Dominik Fachet, Francois Lallemend, Igor Adameyko, Saida Hadjab, and Edouard Hannezo. Theory of branching morphogenesis by local interactions and global guidance. *Nature Communications*, 12(1):6830, 2021.

[2] Edouard Hannezo, Colinda LGJ Scheele, Mohammad Moad, Nicholas Drogo, Rakesh Heer, Rosemary V Sampogna, Jacco Van Rheenen, and Benjamin D Simons. A unifying theory of branching morphogenesis. *Cell*, 171(1):242–255, 2017.

[3] S Lawrence Zipursky and Wesley B Grueber. The molecular basis of self-avoidance. *Annual review of neuroscience*, 36:547–568, 2013.

[4] Elisabeth G Rens, Mathé T Zeegers, Iraes Rabbers, András Szabó, and Roeland MH Merks. Autocrine inhibition of cell motility can drive epithelial branching morphogenesis in the absence of growth. *Philosophical Transactions of the Royal Society B*, 375(1807):20190386, 2020.

[5] Paul M Chaikin, Tom C Lubensky, and Thomas A Witten. *Principles of condensed matter physics*, volume 10. Cambridge university press Cambridge, 1995.

[6] J Van Pelt and RWH Verwer. Topological properties of binary trees grown with order-dependent branching probabilities. *Bulletin of mathematical biology*, 48(2):197–211, 1986.

[7] Ignacio Bordeu, Lemonia Chatzeli, and Benjamin D Simons. Inflationary theory of branching morphogenesis in the mouse salivary gland. *Nature Communications*, 14(1):3422, 2023.

[8] Guilhem Mascré, Sophie Dekoninck, Benjamin Drogat, Khalil Kass Youssef, Sylvain Brohée, Panagiota A Sotiropoulou, Benjamin D Simons, and Cédric Blanpain. Distinct contribution of stem and progenitor cells to epidermal maintenance. *Nature*, 489(7415):257–262, 2012.

[9] Anna M Lilja, Veronica Rodilla, Mathilde Huyghe, Edouard Hannezo, Camille Landragin, Olivier Renaud, Olivier Leroy, Steffen Rulands, Benjamin D Simons, and Silvia Fre. Clonal analysis of notch1-expressing cells reveals the existence of unipotent stem cells that retain long-term plasticity in the embryonic mammary gland. *Nature cell biology*, 20(6):677–687, 2018.

[10] Allon M Klein and Benjamin D Simons. Universal patterns of stem cell fate in cycling adult tissues. *Development*, 138(15):3103–3111, 2011.

[11] Lars Jakobsson, Claudio A Franco, Katie Bentley, Russell T Collins, Bas Ponsioen, Irene M Aspalter, Ian Rosewell, Marta Busse, Gavin Thurston, Alexander Medvinsky, et al. Endothelial cells dynamically compete for the tip cell position during angiogenic sprouting. *Nature cell biology*, 12(10):943–953, 2010.

[12] Magdalena K Sznurkowska, Edouard Hannezo, Roberta Azzarelli, Steffen Rulands, Sonia Nestorowa, Christopher J Hindley, Jennifer Nichols, Berthold Göttgens, Meritxell Huch, Anna Philpott, et al. Defining lineage potential and fate behavior of precursors during pancreas development. *Developmental cell*, 46(3):360–375, 2018.

[13] Byung-Soo Kim, Jun Chen, Talia Weinstein, Eisei Noiri, and Michael S Goligorsky. Vegf expression in hypoxia and hyperglycemia: reciprocal effect on branching angiogenesis in epithelial-endothelial co-cultures. *Journal of the American Society of Nephrology*, 13(8):2027–2036, 2002.

[14] Stefan Van der Walt, Johannes L Schönberger, Juan Nunez-Iglesias, François Boulogne, Joshua D Warner, Neil Yager, Emmanuelle Gouillart, and Tony Yu. scikit-image: image processing in python. *PeerJ*, 2:e453, 2014.

[15] Juan Nunez-Iglesias, Adam J Blanch, Oliver Looker, Matthew W Dixon, and Leann Tilley. A new python library to analyse skeleton images confirms malaria parasite remodelling of the red blood cell membrane skeleton. *PeerJ*, 6:e4312, 2018.

[16] Denis Menshykau, Odyssé Michos, Christine Lang, Lisa Conrad, Andrew P McMahon, and Dagmar Iber. Image-based modeling of kidney branching morphogenesis reveals gdnf-ret based turing-type mechanism and pattern-modulating wnt11 feedback. *Nature communications*, 10(1):239, 2019.
