# Supplementary material for: Self-organized and directed branching results in optimal coverage in developing dermal lymphatic networks
Source: Nat Commun. 2023 Sep 21;14:5878. doi: 10.1038/s41467-023-41456-7 (PMC10514270; doi:10.1038/s41467-023-41456-7)
Supplement: Supplementary file 2 — Description of Additional Supplementary Files [file 41467_2023_41456_MOESM2_ESM.pdf]

Description of Additional Supplementary Files

File Name: Supplementary Movie 1
Description: A stack of a α-LYVE1 stained P2 ear pinna. The stack starts from the ventral surface and ends at the dorsal surface. The last frame shows the maximum projection of the stack. Note the absence of any other LV structures than the deep dorsal LV network. The scale bar is 500μm. The shown stack is a representative of n=2 analyzed P2 ear pinna representing 1 mouse.

File Name: Supplementary Movie 2
Description: A stack of a α-LYVE1 stained P6 ear pinna. The stack starts from the deep dorsal network and ends at the ventral surface. The yellow arrows point at the location where the dorsal network has sprouted to the ventral surface, thus, forming a ventral "tip tree". White arrows point to examples of ventral trees originating from the base of the ear pinna. The scale bar is 500μm. The shown stack is a representative of n=5 analyzed P6 ear pinna, representing 5 mice. The same ear pinna is also shown in Fig. 1B and S1C-E.

File Name: Supplementary Movie 3
Description: Another example stack of a α-LYVE1 stained P6 ear pinna. The stack starts from the deep dorsal network and ends at the ventral surface. The cyan arrow indicates the sprouting of the deep dorsal network to the ventral side, where it forms a collector-like segment, which sprouts to the ventral surface, at the site of yellow arrows, to form "tip trees". White arrows point to examples of ventral trees originating from the base of the ear pinna. The scale bar is 500μm. The shown stack is a representative of n=5 analyzed P6 ear pinna, representing 5 mice.

File Name: Supplementary Movie 4
Description: A stack of a α-LYVE1 stained P6 ear pinna. The stack starts from the deep dorsal network, reaching the dorsal surface. The yellow arrows point at the examples of deep dorsal network sprouting to the dorsal surface. The scale bar is 500μm. The shown stack is a representative of n=5 analyzed P6 ear pinna, representing 5 mice.

File Name: Supplementary Movie 5
Description: A stack of a α-LYVE1 stained P8 ear pinna. The stack starts from the deep dorsal network, reaching the ventral surface. The yellow arrows point at the sites where the dorsal network sprouts and reaches the ventral surface. White arrows point to examples of ventral trees originating from the base of the ear pinna. The scale bar is 500μm. The shown stack is a representative of n=5 analyzed P8 ear pinna, representing 5 mice. The same ear pinna is also shown in Supplementary Fig. 1F.

File Name: Supplementary Movie 6
Description: A stack of a α-LYVE1 stained P8 ear pinna. The stack starts from the deep dorsal network, reaching the dorsal surface. The yellow arrows point at the examples of deep dorsal network sprouting to the dorsal surface. Note that the number of sprout sites and the size of the sub-trees on the dorsal surface are larger than at P6 (video 4). The video starts from the ventral surface. The scale bar is 500μm. The shown stack is a representative of n=5 analyzed P8 ear pinna, representing 5 mice.

File Name: Supplementary Movie 7
Description: A stack of P21 ventral ear dermis stained with α-LYVE1 (gray) and α-VEGFR3 (red). The stack starts at the deeper ventral dermis reaching the superficial ventral dermis. Note that the network is mostly in a single plane. Examples of rare collector segments are indicated with yellow arrows. The depth stamp shows the micrometers in the Z-direction and the scale bar is 1000μm. The shown stack is a representative of n=10 analyzed P21 ear pinna, representing 10 mice.

File Name: Supplementary Movie 8
Description: A high Z-resolution stack of P21 ventral ear pinna dermis stained with α-LYVE1 (red) and α-VEGFR3 (gray). The depth stamp shows the micrometers in the Z-direction and the scale bar is 200μm.

File Name: Supplementary Movie 9
Description: A stack of P21 dorsal ear dermis stained with α-LYVE1 (gray) and α-VEGFR3 (red). The stack starts at the deeper dorsal dermis reaching the superficial dorsal dermis. Note that, in comparison to the ventral dermis (Video 7) the dorsal network contains abundant deep collector vessels (red) indicated with yellow arrows. The depth stamp shows the micrometers in the Z-direction and the scale bar is 1000μm. The shown stack is a representative of n=3 analyzed ear pinna, representing 3 mice.

File Name: Supplementary Movie 10
Description: Exemplary output of a numerical simulation without side-branching (see also Fig. 2D-E). Active tips are in red while the network in black. The thin black circle represents the growing boundary of the ear pinna.

File Name: Supplementary Movie 11
Description: Exemplary output of a numerical simulation with side-branching (see also Fig. 2F-G). Active tips are in red while the network in black. The thin black circle represents the growing boundary of the ear pinna.

File Name: Supplementary Movie 12
Description: Exemplary output of a numerical simulation with side-branching and different initial condition (another tip tree from the edge, see also Supplementary Fig. 6H). Active tips are in red while the network in black. The thin black circle represents the growing boundary of the ear pinna

File Name: Supplementary Software 1
Description: Code for the numerical branching simulations, with exemplary outputs and visualization.
