# Supplementary material for: Self-organized and directed branching results in optimal coverage in developing dermal lymphatic networks
Source: Nat Commun. 2023 Sep 21;14:5878. doi: 10.1038/s41467-023-41456-7 (PMC10514270; doi:10.1038/s41467-023-41456-7)
Supplement: Supplementary file 15 — Supplementary Software 1 [file 41467_2023_41456_MOESM15_ESM.zip › code_submission_final/README.rtf]

Code to simulate lymphatic branching morphogenesis (Ucar et al)Core code:“lymphatic_morphogenesis.py”. Written in Python 3.9, which implement branching and annihilating random walks on an expanding domain with side branching (see also Hannezo et al, 2017 and Ucar et al, 2021 for further details). Outputs:Key outputs are stored in the - “data” sub-folder (positions, angles, ID etc of all particles at different time point of the simulations, appended numbers represent simulation time, from 0 to 500).- “GNF” sub-folder (analysis of the density fluctuations, first column is length scale, second column is average particle number, third column is SD in particle number, appended numbers represent simulation time, from 0 to 500).Visualization:We also include a typical movie output of a simulation as shown in Fig. 5A: - Output_simulations.gifThis output can be generated directly from the “data” subfolder by running a small code Gnuplot code (version 5.4), called as a shell script: ./plotting_trees.sh
