# Supplementary figures and images for: Self-organized and directed branching results in optimal coverage in developing dermal lymphatic networks
Source: Nat Commun. 2023 Sep 21;14:5878. doi: 10.1038/s41467-023-41456-7 (PMC10514270; doi:10.1038/s41467-023-41456-7)

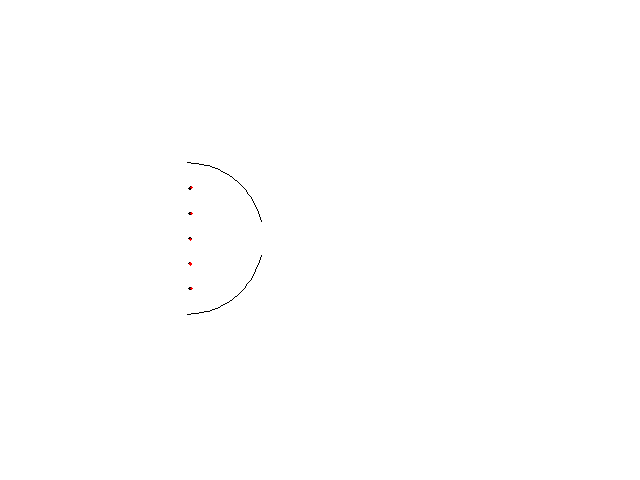

Supplement: Supplementary file 15 — Supplementary Software 1 [file 41467_2023_41456_MOESM15_ESM.zip › code_submission_final/Output_simulations.gif]
