# Supplementary material for: Self-organized and directed branching results in optimal coverage in developing dermal lymphatic networks
Source: Nat Commun. 2023 Sep 21;14:5878. doi: 10.1038/s41467-023-41456-7 (PMC10514270; doi:10.1038/s41467-023-41456-7)
Supplement: Supplementary file 16 — Reporting Summary [file 41467_2023_41456_MOESM16_ESM.pdf]

nature portfolio

Corresponding author(s): Dr. Edouard Hannezo and Dr. Kari Vaahtomeri

Last updated by author(s): Aug 30, 2023

Reporting Summary

Nature Portfolio wishes to improve the reproducibility of the work that we publish. This form provides structure for consistency and transparency in reporting. For further information on Nature Portfolio policies, see our Editorial Policies and the Editorial Policy Checklist.

Statistics

For all statistical analyses, confirm that the following items are present in the figure legend, table legend, main text, or Methods section.

n/a	Confirmed	
☐	☒	The exact sample size (*n*) for each experimental group/condition, given as a discrete number and unit of measurement
☐	☒	A statement on whether measurements were taken from distinct samples or whether the same sample was measured repeatedly
☐	☒	The statistical test(s) used AND whether they are one- or two-sided<br>*Only common tests should be described solely by name; describe more complex techniques in the Methods section.*
☒	☐	A description of all covariates tested
☒	☐	A description of any assumptions or corrections, such as tests of normality and adjustment for multiple comparisons
☐	☒	A full description of the statistical parameters including central tendency (e.g. means) or other basic estimates (e.g. regression coefficient) AND variation (e.g. standard deviation) or associated estimates of uncertainty (e.g. confidence intervals)
☐	☒	For null hypothesis testing, the test statistic (e.g. *F*, *t*, *r*) with confidence intervals, effect sizes, degrees of freedom and *P* value noted<br>*Give P values as exact values whenever suitable.*
☒	☐	For Bayesian analysis, information on the choice of priors and Markov chain Monte Carlo settings
☒	☐	For hierarchical and complex designs, identification of the appropriate level for tests and full reporting of outcomes
☒	☐	Estimates of effect sizes (e.g. Cohen's *d*, Pearson's *r*), indicating how they were calculated

Our web collection on statistics for biologists contains articles on many of the points above.

Software and code

Policy information about availability of computer code

Data collection	Data was collected with Zeis LSM780, 880, or and Andor dragonfly microscope equipped with Zen 2010, Zen2, and Fusion 2.0 softwares, respectively.
Data analysis	FiJi (version 2.0.0 or earlier; NIH), Imaris (version 9.5.1. or earlier; Bitplane), ilastik (1.3.3post2; ilastik.org) , Prism (version 8 or earlier; Graphpad), Microsoft Excel software Mac version (16.66.1. or earlier). Graphs and figures were generated in Adope Photoshop (version 21.1.0) and Adobe Illustrator (version 24.0.3) softwares. Custom codes were written in Python 3.

For manuscripts utilizing custom algorithms or software that are central to the research but not yet described in published literature, software must be made available to editors and reviewers. We strongly encourage code deposition in a community repository (e.g. GitHub). See the Nature Portfolio guidelines for submitting code & software for further information.

Data

Policy information about availability of data

All manuscripts must include a data availability statement. This statement should provide the following information, where applicable:

- Accession codes, unique identifiers, or web links for publicly available datasets
- A description of any restrictions on data availability
- For clinical datasets or third party data, please ensure that the statement adheres to our policy

The Source Data accompanies the manuscript. The data that support the findings of this study are available from the corresponding authors upon request.

Research involving human participants, their data, or biological material

Policy information about studies with human participants or human data. See also policy information about sex, gender (identity/presentation), and sexual orientation and race, ethnicity and racism.

Reporting on sex and gender	N/A
Reporting on race, ethnicity, or other socially relevant groupings	N/A
Population characteristics	N/A
Recruitment	N/A
Ethics oversight	N/A

Note that full information on the approval of the study protocol must also be provided in the manuscript.

Field-specific reporting

Please select the one below that is the best fit for your research. If you are not sure, read the appropriate sections before making your selection.

[x] Life sciences [ ] Behavioural & social sciences [ ] Ecological, evolutionary & environmental sciences

For a reference copy of the document with all sections, see nature.com/documents/nr-reporting-summary-flat.pdf

Life sciences study design

All studies must disclose on these points even when the disclosure is negative.

Sample size	We determined sample-sizes based on the previous experimental observations and similar published experiments in which statistically significant differences were observed (e.g. Zhang et al. 2018, Nat. Comm.; Bovay et al. 2018, J. Exp. Med.; Nurmi et al. 2015, EMBO Mol. Med.).
Data exclusions	No data were excluded.
Replication	All experimental data is described in the article, including replicates. Details of experimental replicates are given in the figure legends. All attempts of replication were successful.
Randomization	Wild-type mice were randomly assigned to different groups. In experiments involving gene deleted mice, allocation of mice into experimental groups was based on genotype and littermate controls were included. Both female and male mice were included in the analyses.
Blinding	For most of the experiments, no blinding was done in the data collection, analysis and quantifications, because the mice were marked for the genotype/treatment, and collected data was quantitative and not influence by investigator's bias. However, segmentation, skeletonization, and measurement of vessel parameters was done in most of the quantified experiments in semi-automated or automated manner.

Reporting for specific materials, systems and methods

We require information from authors about some types of materials, experimental systems and methods used in many studies. Here, indicate whether each material, system or method listed is relevant to your study. If you are not sure if a list item applies to your research, read the appropriate section before selecting a response.

Materials & experimental systems

n/a	Involved in the study	
[ ]	[x]	Antibodies
[x]	[ ]	Eukaryotic cell lines
[x]	[ ]	Palaeontology and archaeology
[ ]	[x]	Animals and other organisms
[x]	[ ]	Clinical data
[x]	[ ]	Dual use research of concern
[x]	[ ]	Plants

Methods

n/a	Involved in the study	
[x]	[ ]	ChIP-seq
[x]	[ ]	Flow cytometry
[x]	[ ]	MRI-based neuroimaging

Antibodies

Antibodies used	rat anti-mouse LYVE1, clone #223322, R&D systems MAB2125; goat anti-VEGFR3, polyclonal, R&D AF743; rabbit anti-collagen IV,

	polyclonal, Abcam Ab6586; chicken anti-GFP, polyclonal, Abcam ab13970; rabbit anti-RFP, polyclonal, Rockland 600-401-379; Alexa Fluor 488 donkey anti-chicken, polyclonal, Jackson laboratories 703-545-155; Alexa Fluor 594 donkey anti-rat, polyclonal, Invitrogen A21209; Alexa Fluor 594 donkey anti-rabbit, polyclonal, Invitrogen A21207; Alexa Fluor 594 donkey anti-goat, polyclonal, Invitrogen A11058; Alexa Fluor 647 donkey anti-rat, polyclonal, Jackson ImmunoResearch 712-606-153; Alexa Fluor 647 donkey anti-goat, polyclonal, Invitrogen A21207.
Validation	The antibodies used in this study were validated for the species and applications by the indicated manufacturers.<br><br>Antibodies used for whole-mount immunostaining in this study:<br>rat anti-mouse LYVE1, R&D systems MAB2125: https://www.rndsystems.com/products/mouse-lyve-1-antibody-223322_mab2125<br>goat anti-VEGFR3; R&D AF743: https://www.rndsystems.com/products/mouse-vegfr3-flt-4-antibody_af743<br>rabbit anti-collagen IV, Abcam Ab6586: https://www.abcam.com/products/primary-antibodies/collagen-iv-antibody-ab6586.html<br>chicken anti-GFP, Abcam ab13970: https://www.abcam.com/products/primary-antibodies/gfp-antibody-ab13970.html<br>rabbit anti-RFP, Rockland 600-401-379: https://www.rockland.com/categories/primary-antibodies/rfp-antibody-pre-adsorbed-600-401-379/

Animals and other research organisms

Policy information about studies involving animals; ARRIVE guidelines recommended for reporting animal research, and Sex and Gender in Research

Laboratory animals	The study involved wild-type and genetically modified mice. The wild-type mice were maintained at C57BL/6 background, Cdh5-CreERT2;Clp24flox/flox mice in C57BL/6 background, Vegfc+/- mice were in ICR (CD1) background, Prox1CreERT2;R26R-confetti in mixed background, and Sox9-Gfp mice in B6/Crl background. The age of the collected and analyzed mice was 1 to 28 days.
Wild animals	The study did not involve wild animals
Reporting on sex	Both female and male mice were used in this study. The data on sex (male/female) was not collected.
Field-collected samples	The study did not involve samples collected from the field
Ethics oversight	All the mice were bred and handled according to the local ethical regulations. Experimental procedures were approved by the Project Authorization Board in Finland (animal license ESAVI/30523/2019 and ESAVI/40857/2022).

Note that full information on the approval of the study protocol must also be provided in the manuscript.
